# Epithelial Markers aSMA, Krt14, and Krt19 Unveil Elements of Murine Lacrimal Gland Morphogenesis and Maturation

**DOI:** 10.3389/fphys.2017.00739

**Published:** 2017-09-26

**Authors:** Alison Kuony, Frederic Michon

**Affiliations:** Developmental Biology Program, Institute of Biotechnology, University of Helsinki Helsinki, Finland

**Keywords:** lacrimal gland, Krt14, Krt19, aSMA, cell proliferation, MET, morphogenesis, branching

## Abstract

As an element of the lacrimal apparatus, the lacrimal gland (LG) produces the aqueous part of the tear film, which protects the eye surface. Therefore, a defective LG can lead to serious eyesight impairment. Up to now, little is known about LG morphogenesis and subsequent maturation. In this study, we delineated elements of the cellular and molecular events involved in LG formation by using three epithelial markers, namely aSMA, Krt14, and Krt19. While aSMA marked a restricted epithelial population of the terminal end buds (TEBs) in the forming LG, Krt14 was found in the whole embryonic LG epithelial basal cell layer. Interestingly, Krt19 specifically labeled the presumptive ductal domain and subsequently, the luminal cell layer. By combining these markers, the Fucci reporter mouse strain and genetic fate mapping of the *Krt14*+ population, we demonstrated that LG epithelium expansion is fuelled by a patterned cell proliferation, and to a lesser extent by epithelial reorganization and possible mesenchymal-to-epithelial transition. We pointed out that this epithelial reorganization, which is associated with apoptosis, regulated the lumen formation. Finally, we showed that the inhibition of Notch signaling prevented the ductal identity from setting, and led to a LG covered by ectopic TEBs. Taken together our results bring a deeper understanding on LG morphogenesis, epithelial domain identity, and organ expansion.

## Introduction

**Figure 1:**
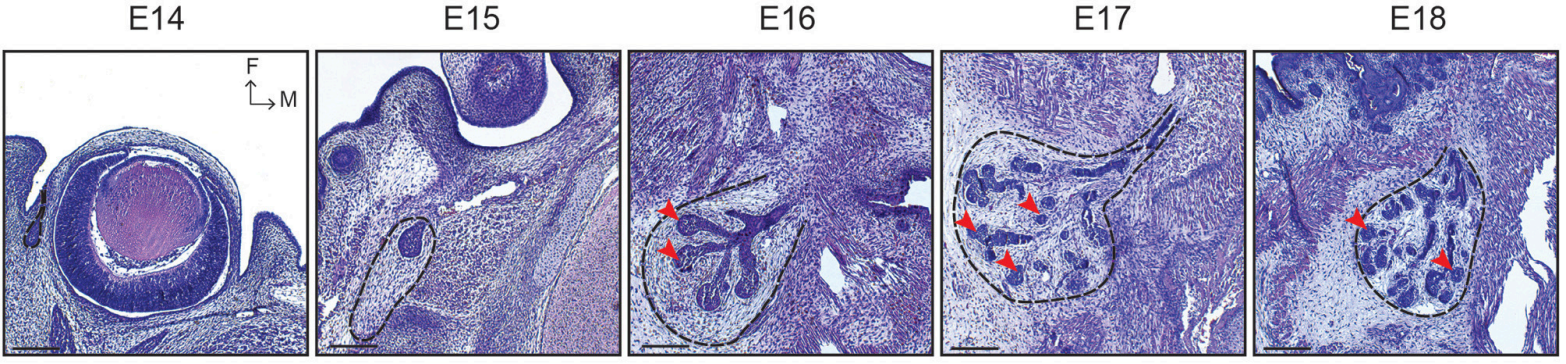
LG morphogenesis from E14 to E18. Histological sections show the LG pre-bud sprouting from the conjunctival epithelium at E14. At E15, the LG epithelial bud elongates and is surrounded by a mesenchymal capsule (black dotted line). Branching morphogenesis occurs from E16 onwards, forming TEBs at the distal extremity of each branch (red arrowheads). Scale bars: 200 μm. M, medial; F, frontal.

The lacrimal gland (LG) is responsible for producing the aqueous component of the three-layered tear film, instrumental for the protection of the eye surface and nutrition of the avascularised cornea (Zieske, 2004). Similarly to other ectodermal organs, the LG originates from an interaction between the epithelium and the underlying mesenchyme (Dhouailly, 2009). At E14, the conjunctival epithelium invaginates at the temporal edge of the eye to initiate LG morphogenesis (Figure 1; Makarenkova et al., 2000). At E15, the epithelial bud and the principal duct have elongated away from the eye, reaching a mesenchymal capsule. From this stage onwards, the epithelial compartment initiates a ductal invasion in the surrounding mesenchyme, similarly as in mammary gland development (Sternlicht, 2006). The first branching events occur at E16, with the formation of TEBs located at the end of each branch. TEBs are composed of a highly organized basal cell layer and of more loosely arranged suprabasal cells. At E17, a second LG lobe emerges from the main duct. This intra-orbital lobe (IOL) is smaller and stays in contact with the eye, while at this stage the larger extra-orbital lobe (EOL) is established in its final location, close to the ear region.

During the murine postnatal period, intense organ development and maturation occur. Around postnatal day (P) 14, the murine eyelid opens, which is associated with the corneal stratification (Zieske, 2004). The growth factors for this process originate from the tear film (Klenkler et al., 2007). This observation demonstrates a change in the tear film composition, reflecting a progressive LG maturation associated with its function.

The mature LG epithelium is composed of three domains, i.e. myoepithelial cells (MECs), acinar, and tubular compartments, which synthesize, modify, secrete and excrete the LG fluid (Katona et al., 2014; Makarenkova and Dartt, 2015). These structures are organized as lobules and lobes (Schechter et al., 2010) and can regenerate after induced injury (Zoukhri et al., 2007, 2008).

Dry Eye Diseases (DEDs) are characterized by an unstable tear film, leading to defective moistening of the eye. DEDs are multifactorial, and affect up to 34% of the population with a higher prevalence in the elderly (Gayton, 2009). DEDs can arise from a defective LG, and result in an impaired vision decreasing the quality of life (for review, Lin and Yiu, 2014) The lack of basic knowledge on LG development, specific cellular mechanisms and cell population identities has impaired the successful development of new therapies restoring LG integrity.

Several signaling pathways are involved in LG morphogenesis and branching regulation (Makarenkova et al., 2000; Dean et al., 2004; Tsau et al., 2011; Chen et al., 2014). Among them, the Notch signaling regulatory network has recently been implicated in early mouse LG development (Dvoriantchikova et al., 2017). Moreover, for the first time, a recent report has demonstrated the implication of microRNAs activity in LG initiation, thus adding to the complex regulation of branched organs morphogenesis (Farmer et al., 2017).

In our study, we used *ex vivo* cultures, transcriptomic analysis, whole mount, and immunohistochemical staining to address LG morphogenesis and subsequent maturation, and try to pinpoint the specificities of this gland compared to others. Here, we used specific epithelial markers, i.e., *Keratin14* (*Krt14*), *Acta2* (encoding for *alpha-Smooth Muscle Actin*, and hereafter referred as *aSMA*), and *Keratin19* (*Krt19*) to follow LG epithelial cell dynamics and glandular tree expansion. Furthermore, we showed that *Krt14*+ and *aSMA*+ cell populations were partially overlapping during LG embryonic development. Interestingly, we found that the myoepithelial cells (MECs) were double positive in the mature LG. We also demonstrated that not only cell proliferation, but epithelial reorganization and mesenchymal-to-epithelial transition supported LG epithelial expansion. By using Krt19 as marker of the luminal cells, we provided evidence that LG patterning was mostly stochastic. Eventually, we established the decisive role of Notch pathway to set the ductal identity in the LG tree.

## Materials and methods

### Animals and tissue processing

All aspects of mouse experiments were approved by the Finnish National Board of Animal Experimentation (ESAVI/1284/04.10.07/2016). The plug day was considered as embryonic day E0 and the date of birth as post-natal day P0. All embryos were staged according to limb morphological criteria.

Wild-type ICR mice and Rosa-R26R reporter transgenic mice [R26R-TdTomato (Madisen et al., 2010), R26R-Confetti (Snippert et al., 2010), and R26R-mT/mG (Muzumdar et al., 2007)] were purchased from Jackson Laboratory, USA. Fucci (Sakaue-Sawano et al., 2008) fluorescent cell cycle reporter mouse strain was used for cell cycle analyses. The reporter lines were crossed with the K14-Cre43 line (Andl et al., 2004), kindly provided by Dr. Sarah Millar, to induce genetic recombination. R26R-mT/mG mice were crossed with aSMA-Cre line (LeBleu et al., 2013), kindly provided by Prof. Kari Alitalo, for recombination tests.

All tissues were fixed with 4% paraformaldehyde (PFA). For E17 and E18 stages, tissues were decalcified with EDTA prior embedding. For histology and immunohistochemistry, tissues were embedded in paraffin and 5 μm thickness sections were used.

### Microarray analysis

LGs were dissected out as previously described (Finley et al., 2014). Total RNA was extracted from whole LGs (mesenchyme and epithelium) of embryonic (E18) and adult (34 weeks of age) animals. E18 samples were composed of at least 10 LGs (5 individuals) pooled together. Biological triplicates for each sample were included in the analysis. The quality and concentration of the extracted RNA was monitored using a nanodrop spectrophotometer (ND-1000, Fisher Scientific). The transcriptomic analysis was performed by the Functional Genomics Unit (FuGU, Helsinki, Finland). Samples were hybridized on Affymetrix GeneChip® Mouse Transcriptome Assay 1.0 (Affymetrix, Santa Clara, CA, USA) microarrays, and data analysis was performed with the Affymetrix Transcriptome Analysis Console (TAC) Software.

### *Ex vivo* culture

LG cultures were established using the Saxén protocol (Munne et al., 2013) and the method described previously (Finley et al., 2014). Culture medium was composed of DMEM/F-12, GlutaMAX supplement (Thermo Fisher Scientific) complemented with 10% FBS, 0.1% Penicillin/Streptomycin and 0.1% Ascorbic Acid (Sigma Chemical Co.). The medium was changed every second day. Dissected LGs were cultured for a maximum of 5 days, at 37°C, in a controlled atmosphere (5% CO_2_).

For Notch pathway inhibition experiments, DAPT 10 μM (Sigma-Aldrich) was added to the medium (Michon et al., 2007). Contralateral controls were supplemented with DMSO (Sigma-Aldrich). Medium and cultures were protected from light. Medium was changed every day. Each experiment was replicated at least five times with over 10 LGs each time.

### Whole mount and immunohistochemistry staining

Both whole mount and immunohistochemistry on slides were performed on PFA-fixed samples. For whole mounts, non-specific staining was blocked by incubating the organs over night at +4°C in a blocking solution (5% donkey/goat serum + 1% BSA in PBS-0.1% Triton). Primary antibodies (see Table below) were diluted in a fresh blocking solution and incubated o/n at +4°C. Subsequently, the glands were incubated with secondary antibodies (see below) o/n at +4°C in PBS-0.1% Triton +1% BSA.

For immunohistochemistry staining on slides, an antigen retrieval step was added to the protocol. Antigen retrieval was performed in 10 mM Na-citric acid (pH 6.0), using an antigen retrieval device (Aptum Biologics Ltd).

Primary antibodies used:

**Table:** 

Antibody	Specie	Company, reference #	WM	IHC
Anti-aSMA	Mouse	Abcam, ab7817	1/100	1/100
Anti-aSMA	Rabbit	Abcam, ab 5694	1/100	1/100
Anti-Krt14	Rabbit	Thermo Fisher Scientific, RB-9020-P	1/100	1/100
Anti-Krt14	Mouse	Abcam, ab7800	_	1/100
Anti-Krt19	Rabbit	Abcam, ab52625	1/100	1/100
Anti-Casp3	Rabbit	Cell Signaling, 9661S	1/400	_
Anti-pH3	Rabbit	Abcam, ab5176	1/100	_
Anti-E-Cadh	Mouse	BD Biosciences, 610182	1/500	1/750
Anti-E-Cadh	Rat	ThermoFisher, 13-1900	_	1/750
Anti-Notch2	Rabbit	Abcam, ab8926	1/100	1/100
Anti-Notch2	Rabbit	ImmunoWay, YC0069	1/200	_

Anti-Notch2 (ImmunoWay, YC0069) was kindly provided by Irene Ylivinkka and Arvydas Dapkunas.

Secondary antibodies used included anti-rabbit AlexaFluor 488 (Life Technologies), anti-mouse AlexaFluor 568 (Life Technologies) and anti-rat AlexaFluor 647 (Invitrogen). Secondary antibodies were diluted at 1/500 for whole mounts and at 1/400 for immunohistochemistry on slides.

In both protocols, the samples were counterstained with Hoechst 33342 (1/2000, Life Technologies) for nuclei staining, and mounted in Vectashield (Vector Laboratories) prior to microscopy visualization.

### Reverse transcription (RT) and multiplex quantitative real time PCR

RNeasy microkit (Qiagen) was used according to the manufacturer's instructions to extract total RNA from dissected LGs of animals ranging from E15 to adult (34w). cDNAs were generated from biological triplicates by using the SuperScript™ III Reverse Transcriptase kit (for RT PCRs, Invitrogen) or the QuantiTect Reverse Transcription Kit (for multiplex PCRs, Qiagen, 205310), according to the provider's recommendations.

Subsequently to cDNA synthesis, reverse transcription PCRs for Aquaporin 1, 5, and 8 were performed using an annealing temperature of 60°C for 40 cycles. One hundred fifty nanograms of total RNA was used for each reaction.

The primer sequences are given in the following table:

**Table:** 

Aqp1-F	CAAGGACAGCTCAGAGTGCA	Aqp1-R	TGTGCAGCTGTGATATGCCA
Aqp5-F	CGCTCAGCAACAACACAACA	Aqp5-R	GAAAGATCGGGCTGGGTTCA
Aqp8-F	ATTTGGAGGGCTGATTGGGG	Aqp8-R	AGGCTCCAGAGATGCTACCA
g-Actin-F	CTGGTGGATCTCTGTGAGCA	g-Actin-R	AGGCAACTAACAACCGATGG

Multiplex qRT-PCRs (CFX96 Touch™ Real-Time PCR Detection System, Bio-Rad) were performed using iTaq universal probe super mix (Bio-Rad, 1725130). Ten nanograms of cDNA were used per reaction. Probe combinations (PrimePCR Probe Assay, Bio-Rad):

Combination 1: GAPDH-Cy5 (qMmuCEP0039581); Krt14-Hex (qMmuCEP0058885); Acta2-Cy5.5 (qMmuCIP0032840); Krt19-FAM (qMmuCIP0033699).
Combination 2: GAPDH-Cy5 (qMmuCEP0039581); Notch2-Hex (qMmuCIP0030263); Hey1-Tex615 (qMmuCEP0057542).
Combination 3: GAPDH-Cy5 (qMmuCEP0039581); Hey1-Tex615 (qMmuCEP0057542); Krt14-Hex (qMmuCEP0058885); Acta2-Cy5.5 (qMmuCIP0032840).

Gene expression levels were normalized using *GAPDH* expression levels.

### Imaging and data analysis

Bright field organ morphology was imaged using a Zeiss Lumar stereomicroscope. Immunofluorescence confocal imaging was performed using a Leica TCS SP5 and SP8 confocal microscopes.

Images were analyzed and quantitative measurements performed with Imaris 8.4.1 (Bitplane) software. For the cell cycle analyses with the Fucci mouse line, only the cells that were distinctly identified as either G1 (red), or S/G2/M (green) were included. Proliferation ratios were obtained by dividing the number of green cells by the number of (green + red) cells.

Student *t*-test (paired, two-tailed) was used for statistical analysis. All statistical analyses were performed on biological triplicates. For cell cycle study, a total of 9 TEBs from 3 LGs were used for each developmental stage; for bifurcations analysis, at least 3 LGs of each stage were analyzed. For *aSMA* expression in Notch inhibition study, biological triplicates (at least 3 cultured LGs per condition and per time point) were used. Moreover, technical triplicates were used to validate the experiments.

Images were processed with Photoshop CC 2017 and Illustrator CC 2017 software (Adobe Systems).

## Results

### *Krt14* and *aSMA* expression depict three cell populations in the forming LG

Previous studies reported the expression of *Krt14* and *aSMA* in the embryonic and adult LG (Dean et al., 2004; Hirayama et al., 2016). However, their embryonic expression pattern have not been described so far. Therefore, we focused on these two genes to characterize LG epithelium morphogenesis. By using qRT-PCR, we demonstrated that *Krt14* and *aSMA* expression level constantly increased from E15 to 34 weeks of age (Figure S1A).

**Figure 2:**
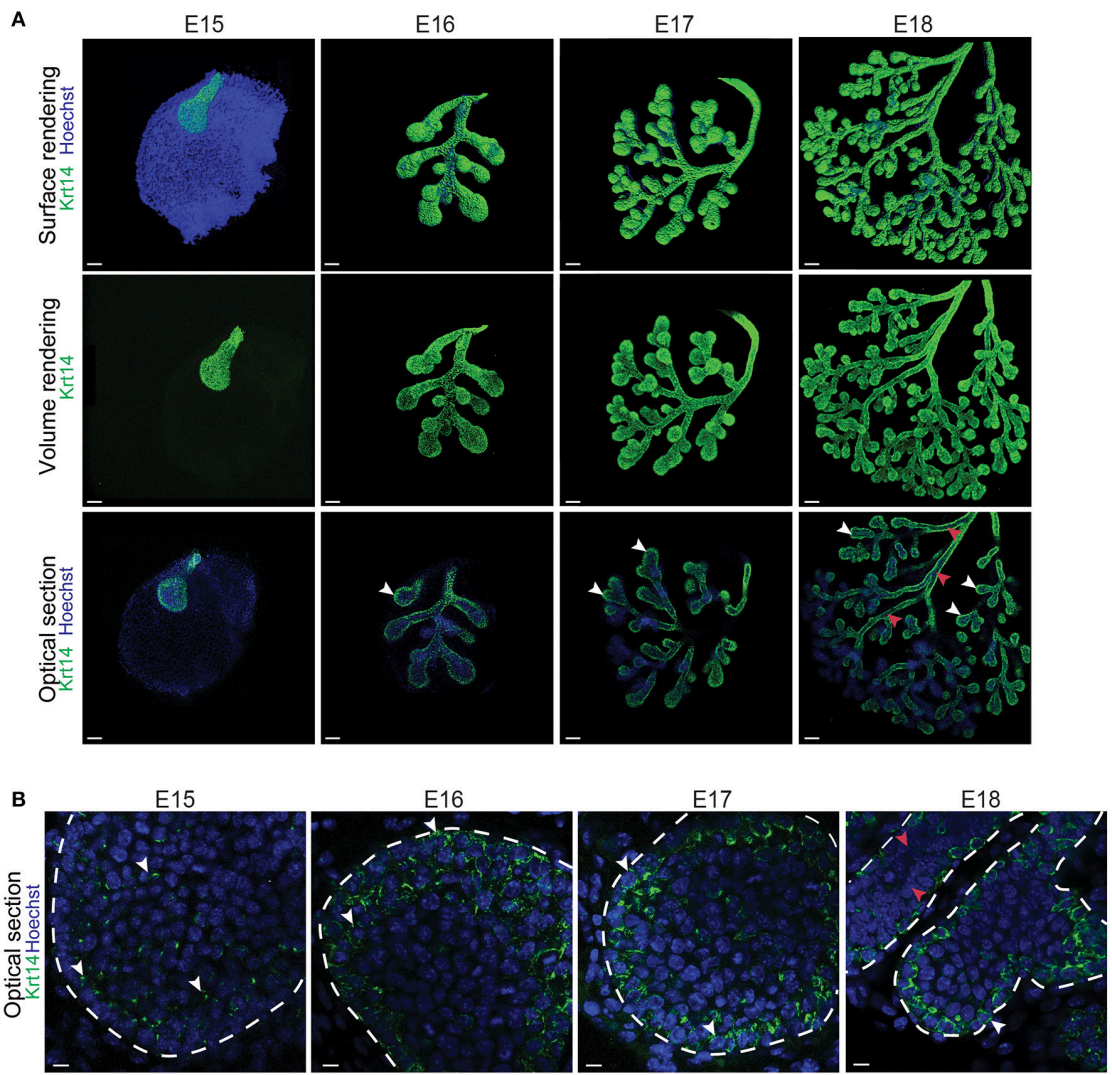
*Krt14*, broadly expressed in the LG epithelial bud, gets restricted to the basal cell layer during branching morphogenesis. **(A)** Whole-mount immunostaining show the localization of Krt14 from E15 to E18. The 3D surface renderings demonstrate the presence of Krt14 at the surface of the LG epithelium at all stages. Optical sections display the enrichment of *Krt14*+ cells in the most basal cell layers (white arrowheads), compared to the suprabasal cell layers that are mostly *Krt14* negative (red arrowheads). **(B)** Close-up visualizations show Krt14 repartition within the epithelial cell layers. At E15, *Krt14* is broadly expressed in the epithelial bud. Then, from E16 to E18, Krt14 is localized in the external layers of the whole epithelial compartment, including the basal cell layer (white arrowheads). However, Krt14 is not present in the inner layer of the ducts (red arrowheads). Scale bars: **(A)** 100 μm; **(B)** 20 μm.

To further analyse the localization of *Krt14*+ and *aSMA*+ cell populations during LG morphogenesis, we studied Krt14 and aSMA patterns using immunofluorescence staining and confocal microscopy. At E15, Krt14 was broadly found in the most external cell layers of the bud epithelium, and not only localized in the basal cell layer (Figures 2A,B). Interestingly from E16 to E18, Krt14 pattern appeared more restricted to the basal cell layer of both TEBs and branches (Figure 2B). In addition, our observations of Krt14 pattern suggested that the luminal cells of the duct were *Krt14*-negative at E18 (Figure 2B).

**Figure 3:**
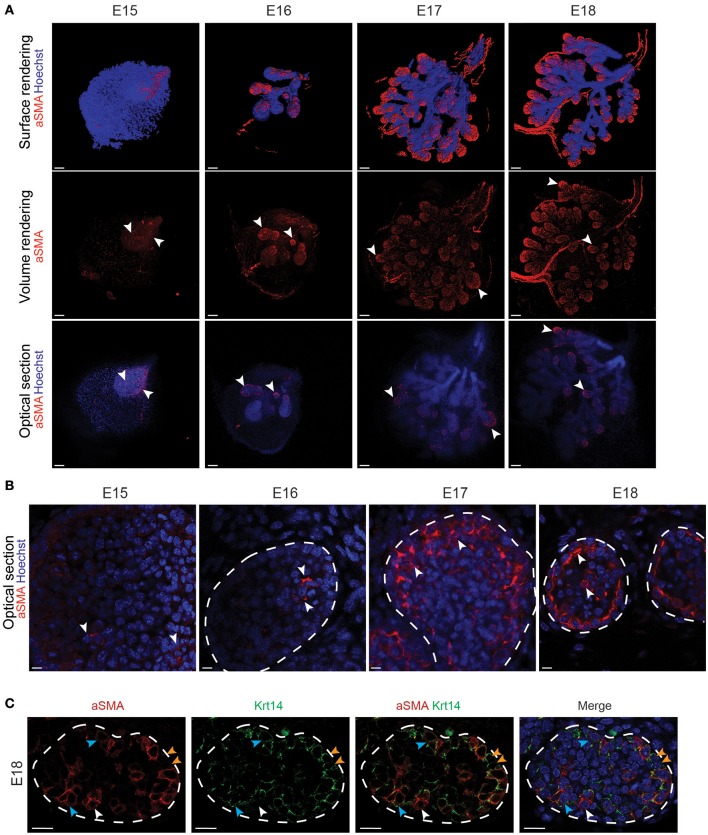
*aSMA* is expressed in the basal cell layer and partially overlaps with *Krt14* expression during LG epithelial compartment morphogenesis. **(A)** Whole-mount immunostaining show the localization of *aSMA*+ cell population from E15 to E18. The 3D surface renderings demonstrate the presence of aSMA mainly in the forming TEBs. Optical sections confirm the restriction of the *aSMA*+ cell population to the TEBs (white arrowheads). The ducts remain *aSMA* negative. **(B)** Close-up visualizations show aSMA repartition within the epithelial cell layers. At E15, *aSMA* is scarcely expressed in the epithelial bud. *aSMA* expression increases along with branching morphogenesis, and gets progressively restricted to the basal layer of the TEBs from E16 onwards (white arrowheads). **(C)** A close-up on the TEBs at E18 shows that aSMA and Krt14 localization partially overlap and define three cell populations in the embryo: *aSMA* single positive cells (white arrowheads), *Krt14* single positive cells (blue arrowheads), and double positive cells (orange arrowheads). Scale bars: **(A)** 100 μm; **(B,C)** 20 μm.

In contrast, *aSMA*+ cells were scattered throughout the bud epithelium at E15 (Figures 3A,B). Later on, *aSMA*+ cells were progressively restricted to the basal cell layer of the TEBs from E16 to E18 (Figures 3A,B). A close-up visualization at E18 revealed a partial overlap of the *Krt14*+ and *aSMA*+ cell populations (Figure 3C). Due to this incomplete overlap, we found three cell populations in the E18 TEBs, namely the *Krt14*+ cells, the *aSMA*+ population and the double positive *Krt14*+;*aSMA*+ cells (Figure 3C).

To visualize the involvement of the *aSMA*+ cells and *Krt14*+ cells during LG morphogenesis, we crossed the aSMA-Cre (LeBleu et al., 2013) and K14-Cre43 (Andl et al., 2004) lines with reporter mouse strains.

**Figure 4:**
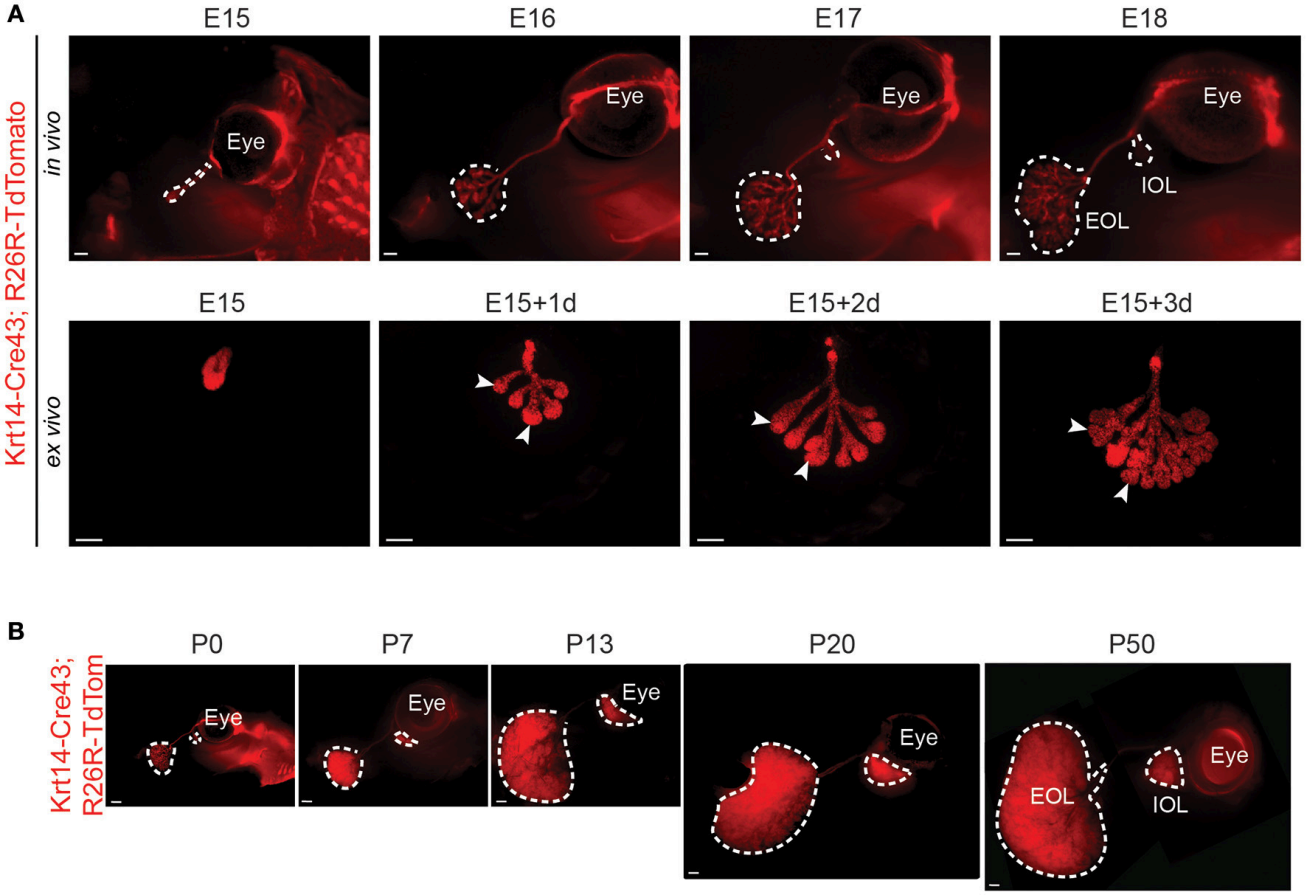
K14-Cre43 transgenic line allows the visualization of embryonic and postnatal LG epithelial morphological changes. K14-Cre43;R26R-Tdtomato mouse strain allows the visualization of LG epithelial compartment morphogenesis. **(A)** LG epithelial morphological changes in embryonic stages. At E15, the LG appears as a non-branched epithelial bud. Branching occurs at E16 and from E17 onwards, the IOL starts to develop at the proximal side of the main duct. *In vivo* LG development (E15 to E18) is comparable to *ex vivo* development (E15 to E15+3 days of culture). Arrowheads point out TEBs. **(B)** External views of *in vivo* LG development from P0 to P50. LG epithelium undergoes a rapid expansion between P7 and P13, during the eyelid opening period, and continues to grow until adulthood. Scale bars: **(A)** 200 μm; **(B)** 500 μm. IOL, Intra-orbital lobe; EOL, Extra-orbital lobe.

Unfortunately, the aSMA-Cre transgenic line displayed a very low recombination level in the LG (Figure S2). As a result, it was impossible to use it for any genetic fate mapping experiment. Therefore, we took advantage of the K14-Cre43 mouse strain, reported to have a high recombination efficiency in various ectodermal organs (Jussila et al., 2015; Shirokova et al., 2016). It allowed the visualization of the LG epithelial morphological changes from E15 to E18 (Figure 4A). As expected from the *Krt14*+ cell localization (Figure 2), the *Krt14*+ cells and their progeny outlined perfectly the branching morphogenesis. Moreover, by comparing cultured LGs to the ones pictured *in situ*, we saw that LG development was similar *in* and *ex vivo* (Figure 4A), providing a great tool for microenvironment modulations.

By following LG postnatal expansion, we found that the epithelial compartment continued its growth until around P50 (Figure 4B). We investigated the aSMA and Krt14 localization in postnatal LG, and noticed that the MECs, recognizable by their long processes, expressed both *aSMA* (as previously reported, Makarenkova and Dartt, 2015) and *Krt14* (Figure S3A). No other cells in the acini expressed any of these markers. Krt14 was also found in the basal cell layer of the ducts. The postnatal continuity of LG morphogenesis went along with a switch in Aquaporins expression, known to be responsible for the tear film composition (Schey et al., 2014). While *Aquaporin1* expression decreased after the embryonic development, *Aquaporin8* was greatly enriched in postnatal LG (Figure S3B).

Our results showed that aSMA and Krt14 can be used as embryonic markers for basal epithelial cell layer of the forming LG. Moreover, the presented data confirmed the continuity of LG formation in postnatal stages. However, the mechanisms involved in the LG growth remained elusive.

### Cell proliferation fuels early LG epithelium expansion

**Figure 5:**
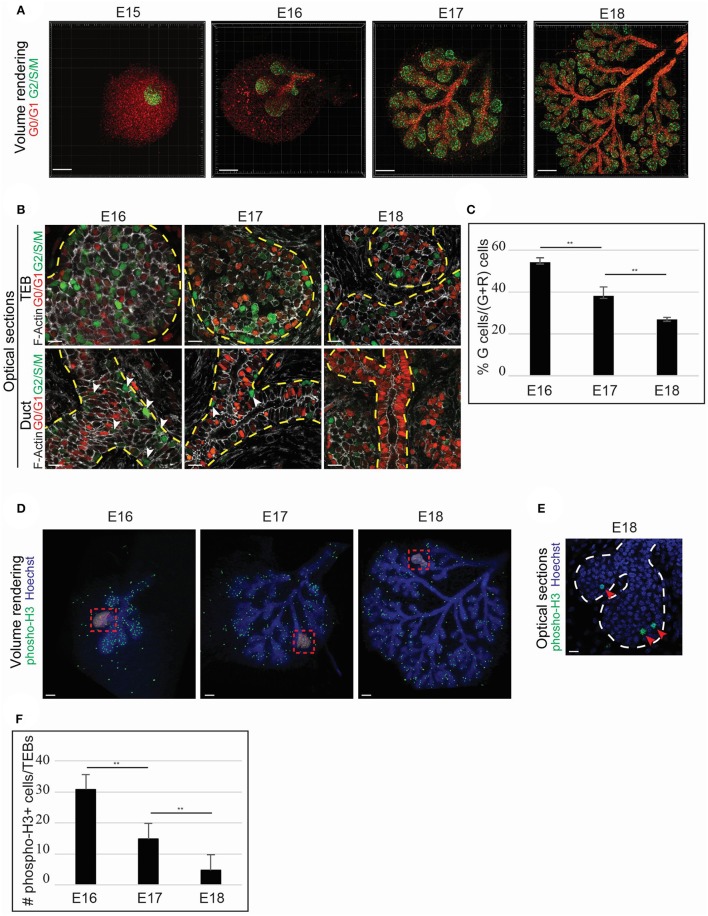
TEB cell proliferation fuels LG epithelial morphogenesis. **(A–C)** The Fucci system allows the visualization of cells in G0/G1 (nuclear red) and S/G2/M (nuclear green) phases and is used to monitor changes in the proliferation status during LG morphogenesis. **(A)** Volume rendering of E15 to E18 Fucci LGs. Proliferation occurs mainly in the epithelial compartment. At E15, the LG epithelial bud depicts majority of cells in G2/S/M. When branching morphogenesis starts (from E16 onwards), the ductal compartment reveals a higher number of G1/G0 cells, while the TEBs are mostly undergoing proliferation. **(B)** Close-up on optical sections. The TEBs show proliferative cells both in basal and suprabasal layers in all the studied stages. However, majority of the cells in the ductal compartment are in G0/G1. At E16 and E17, some cells in G2/S/M can be observed in the ducts (white arrowheads), but close to no cells are proliferating in the ductal compartment at E18. **(C)** The quantification of fluorescent cells in the TEBs demonstrates a clear diminution of the proliferation from E16 to E18. At E16, 54.5% of the cells are proliferating. Proliferation decreases to 38.2% at E17, and to 27.2% at E18. **(D–F)** Phospho-Histone3 whole mount immunostaining display a similar trend in the LG proliferation status from E16 to E18. **(D)** Volume renderings show that phospho-Histone3 positive cells are mainly localized in the epithelial compartment. Insets give an example for the quantification analysis: red dots (false color) correspond to each phospho-Histone3 positive nuclei. **(E)** Close-up on optical section. TEBs shows proliferating cells at E18. **(F)** Similarly to the Fucci analysis, the quantification of phospho-Histone3 positive cells demonstrates a decrease in the TEB proliferative state along with branching morphogenesis. At E16, about 30 cells/TEBs on average are phospho-H3 positive. The number of phospho-H3 positives cells decreases to <15 cells/TEBs at E17, and to 5 cells/TEBs at E18. Scale bars: **(A)** 200 μm; **(B,E)** 20 μm; **(D)** 100 μm. ^**^p < 0.001 were considered as statistically significant (Student's *t*-test). Error bars represent standard deviations (*n* = 9 TEBs/time point). Dotted lines delimitate epithelial regions.

To comprehend the cellular mechanisms involved in LG enlargement, we focused on identifying possible proliferative zones. Therefore, we used the Fucci reporter mouse line (Sakaue-Sawano et al., 2008), in which cells express a green fluorescence in S/G2/M cell cycle phases, and red fluorescence in G0/G1 phases. The cells of the epithelial bud expressed green fluorescence, reflecting a global and unpatterned proliferation at E15 (Figure 5A). However, after the first branching event, the proliferation gradually decreased and seemed more localized in the acinar compartment (Figure 5A). We analyzed the cell cycle state of both the acinar and ductal compartment during branching morphogenesis, and detected proliferating cells in TEB basal and suprabasal cell layers (Figure 5B). Although some of the TEB cells remained in an active cell cycle throughout LG morphogenesis, the ductal cells progressively exited the cell cycle along with LG morphogenesis. By E18, almost no ductal cells were proliferating anymore (Figure 5B). As we noted a decrease of the green fluorescence in the TEBs, we quantified the proliferating cells in the TEBs from E16 to E18. While at E16, about 55% of the TEB cells were proliferating, by E18, only 27% of the cells were still in an active proliferative state (Figure 5C). This observation reflected the progressive decrease and restriction of cell proliferation during LG morphogenesis. We confirmed this gradual restriction of proliferation by quantifying the phosphorylated-Histone3+ cells (pH3), epigenetic modification found only in M phase cells (Hans and Dimitrov, 2001; Figures 5D–F). Nevertheless, we showed the continuation of LG growth after birth (Figure 4B). The general decrease of cell proliferation we observed seemed conflicting with the postnatal expansion of the LG tree. Therefore, we investigated other mechanisms that could support LG growth.

### Epithelial reorganization and MET assist LG glandular tree expansion

**Figure 6:**
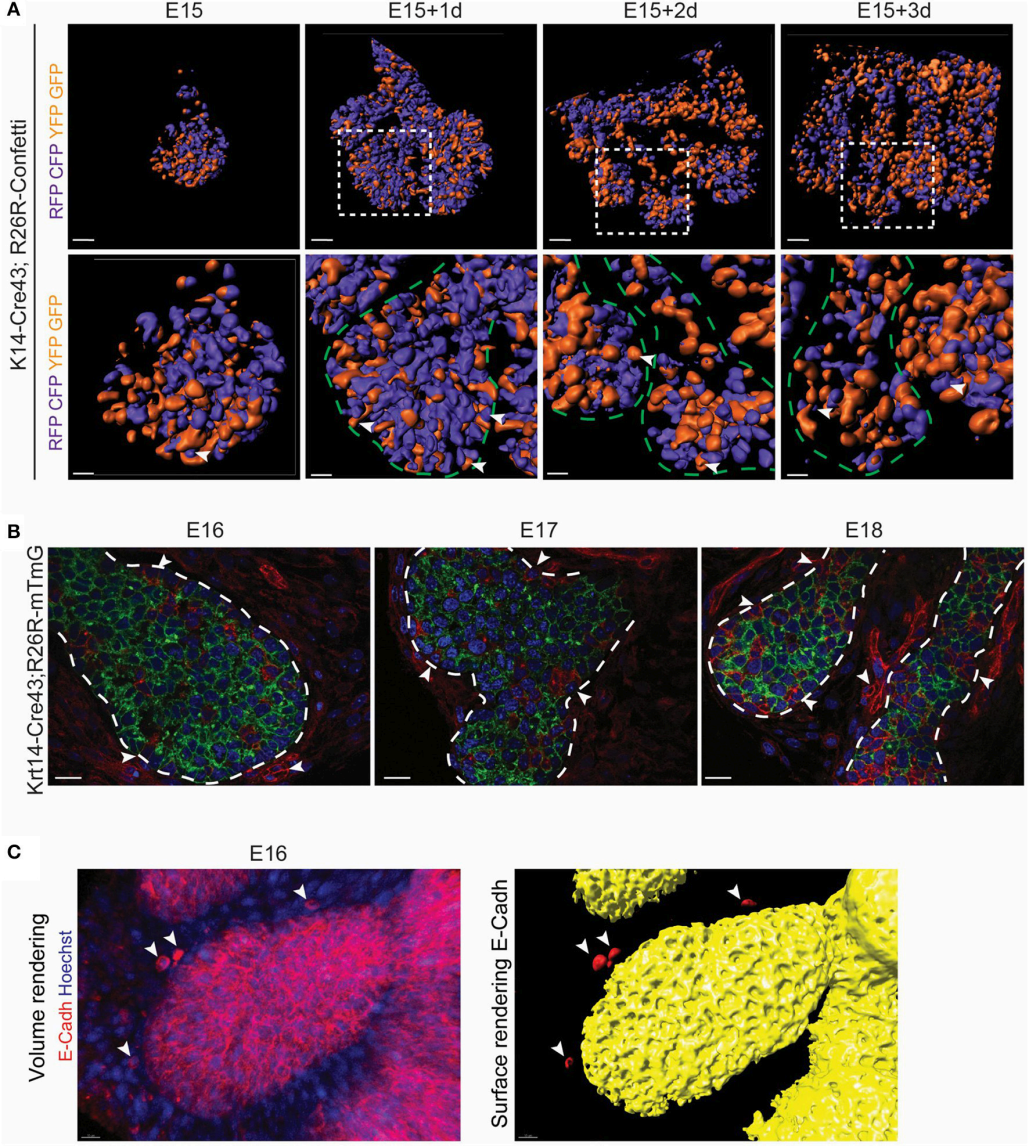
LG branching morphogenesis involves epithelial cell rearrangement and MET. **(A)** Surface renderings of K14-Cre43;R26R-Confetti LG after *ex vivo* cultures show the contribution of *Krt14*+ progeny during branching morphogenesis. RFP and/or mCFP clones are merged into the violet population. YFP and/or GFP clones are pooled into the orange domain. Violet and orange domains allow the visualization of intercalated *Krt14*+ clones from different origins. At any time point, single *Krt14*+ cells of one origin can be observed in the middle of a domain of the other origin (white arrowheads). Insets show the magnified region in the lower panel. **(B)** K14-Cre43;R26R-mT/mG crossing leads to the visualization of *Krt14*+ cells and their progeny in green, and the *Krt14*-negative population and its progeny in red, from E16 to E18. Optical sections depict clusters of mesenchymal cell (red cells, white arrowheads) close to the epithelial surface, as well as in the epithelial compartment itself. The number of red cells within the epithelial compartment increases from E16 to E18. **(C)** At E16, a volume rendering of E-Cadherin whole mount immunostaining. A close-up on a TEB shows *E-Cadherin*+ cells close to the epithelial surface (white arrowheads). Scale bars: **(A)** upper panel: 80 μm; lower panel: 20 μm; **(B)** 20 μm; **(C)** 15 μm. Dotted lines delimitate epithelial regions.

The expansion of a non-proliferative epithelium can result from (i) spreading of a cell layer by change of cell shape, (ii) cellular intercalation or (iii) convergent extension (for review, Keller et al., 2008). During convergent extension, cell rearrangement involves cell intercalation, and lessening of cell layers. This mechanism, in which epithelial cells change layers, can be an advantage for the fast expansion of an epithelial domain. Notably, epithelial rearrangement has been shown to be involved in branching morphogenesis (for review, Wang et al., 2017). To investigate the involvement of this process during LG expansion, we took advantage of the R26R-Confetti reporter mouse line (Snippert et al., 2010). Unlike for a clonal analysis, we used a constitutive Cre (K14-Cre43) to label as many epithelial cells as possible. Upon continuous recombinase activity, cells can be sorted into two populations, depending on the first recombination event (Figure S4A): *GFP* and/or *YFP* expressing cells, and *RFP* and/or *CFP* expressing cells (Figure S4B). Therefore, we separated epithelial cells into two domains: the orange cell domain (*GFP*+ and/or *YFP*+ cells), and the violet cell domain (*RFP*+ and/or *CFP*+; Figure S4C). Surface renderings of *ex vivo* cultures revealed a highly intermingled epithelial population, composed of mix of the two domains during LG morphogenesis. We detected single cells of one origin in the middle of a domain of the other origin. This observation would be incompatible with a simple proliferation-based territory expansion, and hinted toward an expansion of the epithelium via convergent extension (Figure 6A).

Interestingly, a few cells did not display any fluorescence with the Confetti reporter, already at E15 (data not shown). We made similar observations with K14-Cre43;R26R-TdTomato at E17 (Figure S5A) and with K14-Cre43;R26R-mT/mG at E16, E17 and E18 (Figure 6B). We hypothesized that these unlabeled cells could arise from *Krt14* negative origin, or from cells escaping the recombination. To rule out a low efficiency of the K14-Cre43 recombinase, we localized *Krt14*+ cells in the K14-Cre43;R26R-mT/mG embryos (Figure S5B). Some of the mTomato+ cells (not recombined) were Krt14-negative, directing us toward a non-epithelial origin. Moreover, we detected clumps of mesenchymal cells in close contact to the acinus and ductal epithelium (Figure 6B). As cell morphology seemed to indicate a possible mesenchymal cell intercalation within the epithelial compartment (Figure S5B), we analyzed *E-Cadherin* expression, as E-Cadherin is known to act as a mesenchymal to epithelial (MET) effector (Vanderburg and Hay, 1996). Notably, we detected mesenchymal cells at close proximity to the LG epithelium that presented E-Cadherin at their membrane at E16 (Figure 6C). This observation could indicate the involvement of MET during LG morphogenesis, as previously suggested (Dean et al., 2004).

Collectively, we showed that LG epithelium growth is primarily fuelled by cell proliferation in the acini. However, epithelial cell rearrangement and MET might contribute to the ductal tree expansion. Nonetheless, the luminal domain morphogenesis process is still elusive.

### Tubulogenesis engages apoptosis and *Krt19*+ cells

**Figure 7:**
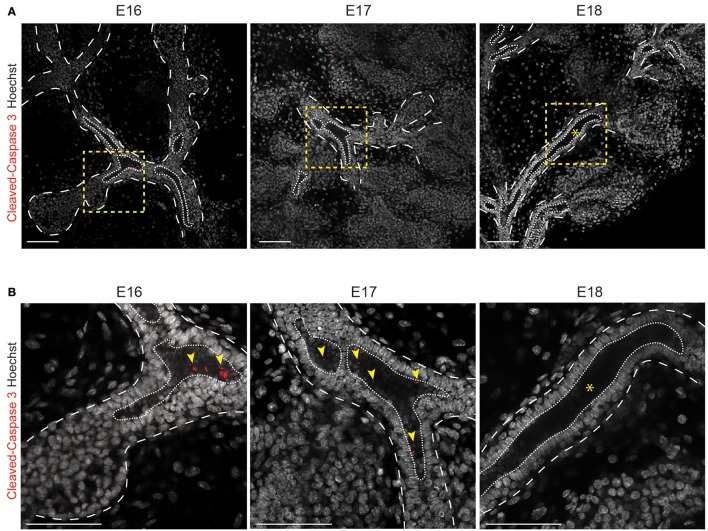
Cleaved-Caspase3+ cells are detected during lumen formation in the ducts. Optical sections of whole mount immunostaining for Cleaved-Caspase3 show a part of the forming LG **(A)** and a close-up **(B)**. Apoptotic cells (red) are observed in the forming lumen from E16 to E18. At E16 and E17, cell death (yellow arrowheads) is observed in the microlumens. By E18, the microlumens fuse (asterisks) and only rare apoptotic cells are still observable in the lumen of the ducts. Dotted line outlines the branches, and the lumen. Insets show the magnified region in the lower panel. Scale bars: 100 μm.

Tubulogenesis is associated with lumen formation, which can be dependent (Wells and Patel, 2010) or independent (Nedvetsky et al., 2014) of cell death. Therefore, we investigated if apoptosis was involved in LG lumen formation. In the same way as in salivary gland duct development (Nedvetsky et al., 2014), we observed the formation of microlumens at E16, concomitantly to the first apoptotic events (Figure 7). This phenomenon peaked around E17, the microlumens joining to form a continuous lumen. By E18, apoptotic cells were close to absent and the lumen was already formed in the larger ducts.

**Figure 8:**
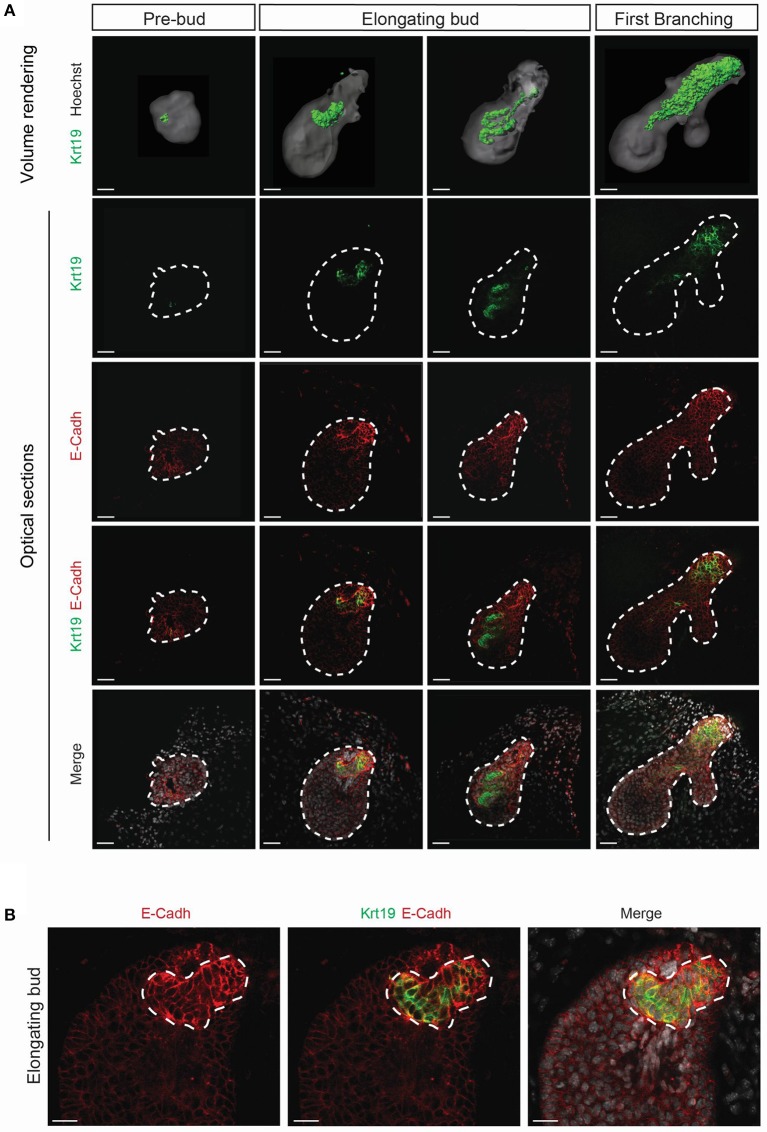
*Krt19* is expressed in the presumptive ductal territory. 3D surface renderings and confocal images of whole mount immunostaining show *Krt19* expression at E15. **(A)** E15 LGs were cultured and collected from E15 to E15+8 h and staged according to morphological criteria until the first branching event. *Krt19* is expressed in a restricted area of the pre-bud. Subsequently, *Krt19*+ cells delineate a 3D spring-like conformation in the elongating bud. After the first branching event, *Krt19*+ domain is extended and more diffuse. **(B)** A close-up on the forming 3D shape reveals an accumulation of E-Cadherin in the *Krt19*+ cells. Scale bars: **(A)** 50 μm; **(B)** 30 μm.

**Figure 9:**
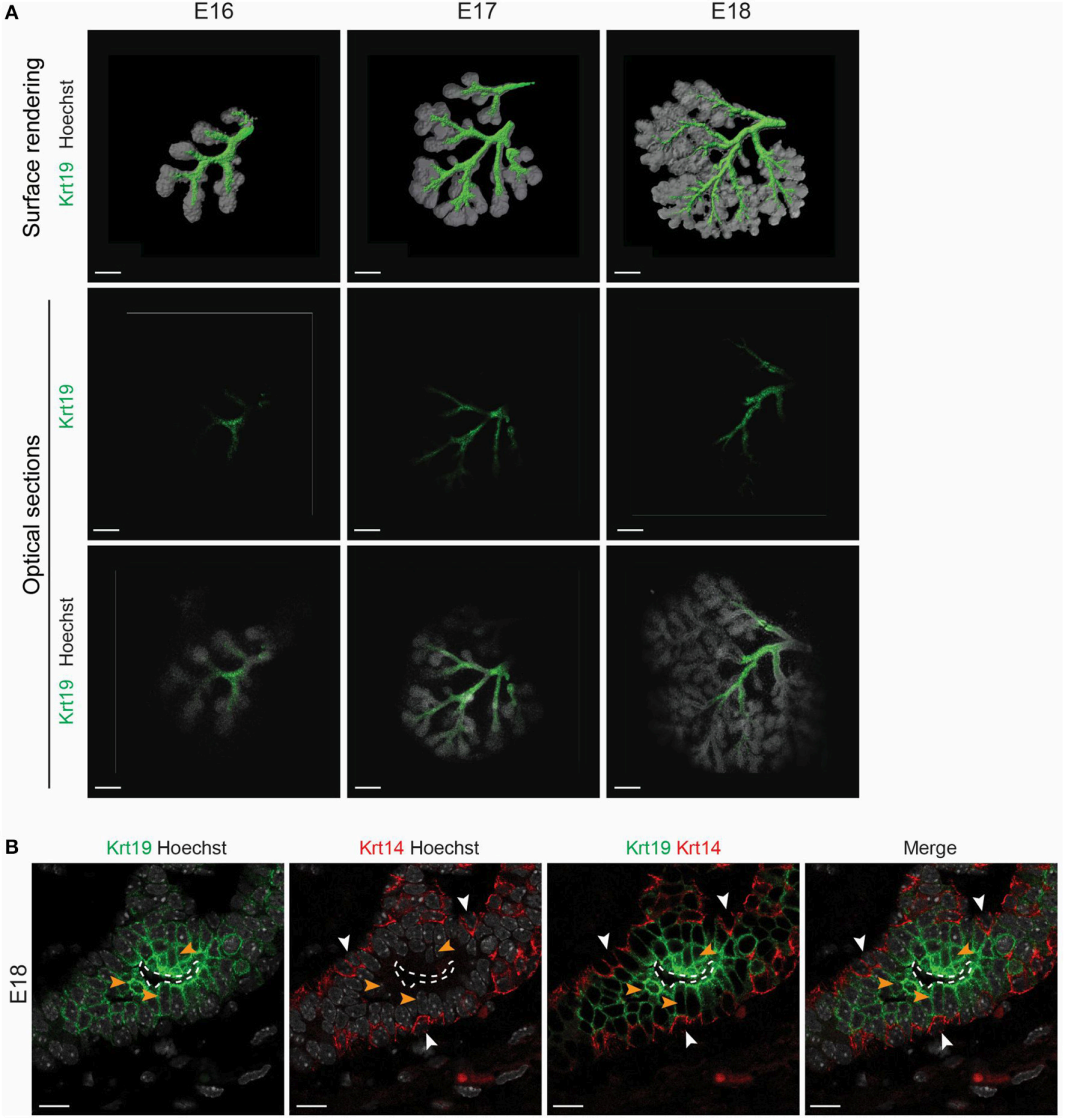
*Krt19* is expressed in the luminal side of the forming ducts. **(A)** 3D surface renderings and confocal images of whole mount immunostaining show Krt19 localization during branching morphogenesis. From E16 to E18, Krt19 is found in the ductal region. **(B)** Immunohistochemistry staining at E18 reveals *Krt19* expression in the luminal cells, and *Krt14* in the basal cells. Scale bars: **(A)** 200 μm; **(B) 10** μm. Dotted line outlines the lumen. White arrowheads (*Krt14*), orange arrowheads (*Krt19*).

*Krt19* has been reported to be expressed in the salivary gland ductal cells (Ogawa et al., 2003), but has never been reported in LG development context. We performed qPCR analysis and found an increase in *Krt19* expression during embryonic LG morphogenesis (Figure S1B). Therefore, we followed Krt19 pattern by immunofluorescence staining and confocal microscopy from E15 to E18 (Figures 8, 9).

Interestingly, we found Krt19 in the LG epithelium at E15, prior to any duct formation. We used short-term *ex vivo* cultures to study this phenomenon (Figure 8A). From the 30 glands dissected at E15 analyzed, around 17% (5 glands) presented a pre-bud phenotype, consisting in a very round LG. About 67% (20 glands) presented an elongated bud, and roughly 17% (5 glands) were already displaying the formation of the first branching event. This transition from pre-bud to the first branching event recapitulated what we observed during LG formation *in vivo*. While Krt19 was seen in a very small and restricted domain in the pre-bud stage, upon bud elongation, *Krt19*+ domain transformed into a 3D spring-like shape. This 3D conformation was transient and rapidly extended within the epithelium domain during the initiation of the branching morphogenesis. Moreover, the *ex vivo* results (Movie 1) were supported by similar observation *in vivo* (Movie 2), ruling out a possible artifact from the *ex vivo* cultures. Interestingly, *E-Cadherin* seemed to be more expressed around the spring-like shape (Figure 8B). This observation might reflect the importance of cell-cell contacts in the *Krt19*+ cells during the transition from E15 to E16.

Subsequently, we investigated the Krt19 localization during the rest of LG morphogenesis. From E16 to E18, Krt19 was detected in the forming ducts (Figure 9A), outlining the progressive formation of the LG tree. A closer observation at E18, when the ducts are already well-extended and the lumen formed, revealed *Krt14*+ cells in the ductal basal cell layer, and *Krt19* expression in the luminal cells (Figure 9B). Notably, this ductal organization remained in all stages of the maturing LG (Figure S6).

**Figure 10:**
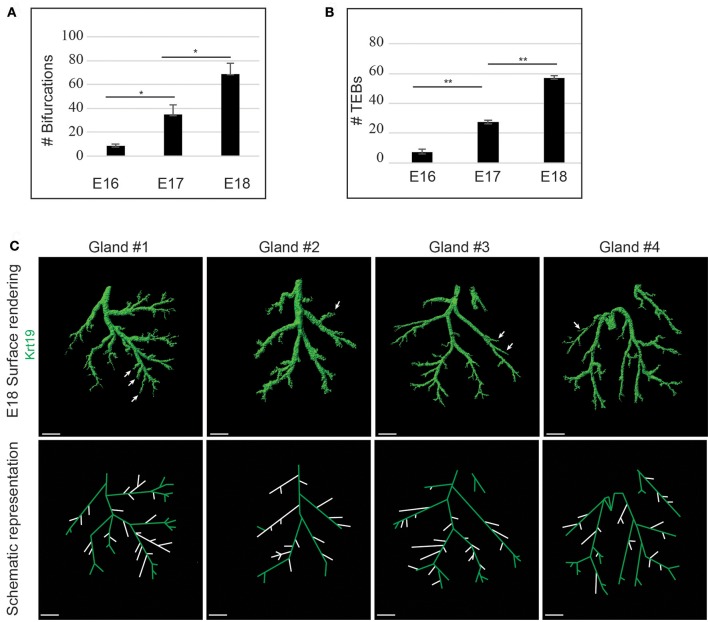
LG branching morphogenesis appears linear but stochastic. **(A,B)** Quantifications show a linear increase of both the amount of bifurcations **(A)** and TEBs **(B)** during branching morphogenesis, from E16 to E18. **(C)** 3D surface renderings and schematic representations of Krt19 whole mount immunostaining of E18 LGs. Each gland exhibits a different location of lateral branches, demonstrating a partly stochastic branching pattern. Arrows and white branches (on the schematic views) highlight lateral branches. Scale bars: **(C)** 200 μm. ^*^p < 0.01 and ^**^p < 0.001 were considered as statistically significant (Student's *t*-test). Error bars represent standard deviations (*n* = 3 LGs per time point).

We took advantage of this marker to analyse further the LG branching process. We quantified both the number of bifurcations (Figure 10A) and the number of TEBs (Figure 10B) between E16 and E18. Despite the decrease of cell proliferation, the formation of new ducts and TEBs seemed to follow a linear increment, reflecting LG morphogenesis regulated pace. Interestingly, by comparing four E18 LG trees, we realized that the junction pattern differed from one gland to another (Figure 10C). Two types of branch formation are described in a glandular tree, i.e. terminal bifurcation (a TEB splitting and forming two growing branches), or lateral branching (a new branch forming from an established one; for review, Affolter et al., 2009). At E18, both lateral branching and terminal bifurcations could be observed in all the studied glands. Notably, the positions of terminal bifurcations and lateral branches seemed to be stochastic, rather than predetermined, giving the tree final shape a large pattern variability.

The formation of lateral branches requires a degree of plasticity from the pre-established ductal cells in order to form a new sprouting TEB (Watanabe and Costantini, 2004). Therefore, we investigated the molecular regulation of the duct identity.

### Notch pathway regulates the ductal domain identity

Notch1 has recently been associated with branching morphogenesis in the LG context (Dvoriantchikova et al., 2017). Moreover, Notch signaling has been linked to the maintenance of suprabasal cells in the salivary gland TEBs (Garcia-Gallastegui et al., 2014), and to cell differentiation in other branched organs, such as lung and liver (Tsao et al., 2008; Falix et al., 2014).

**Figure 11:**
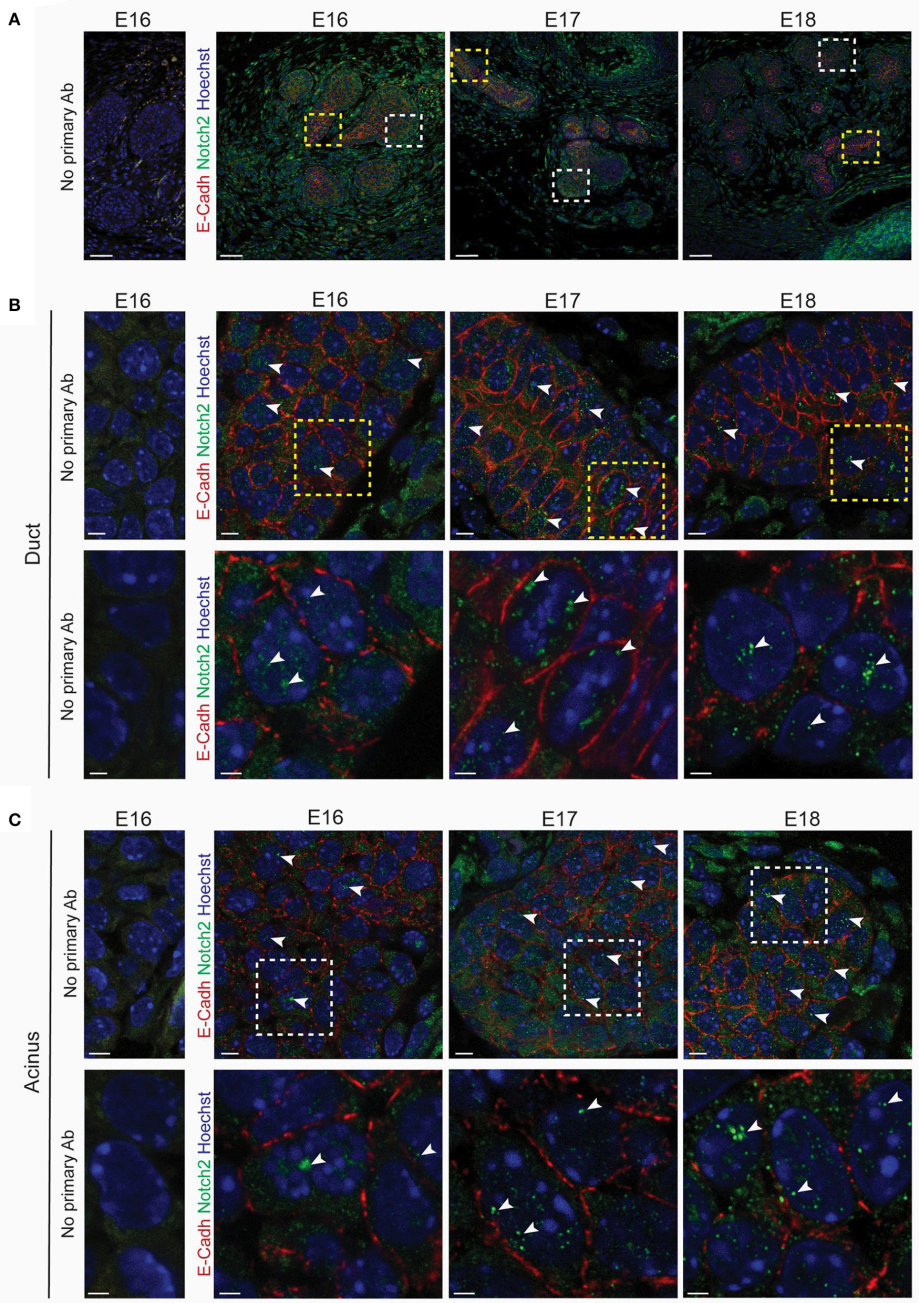
*Notch2* is expressed in developing LG. **(A–C)** Immunohistochemistry staining from E16 to E18. Optical sections show Notch2 pattern during LG morphogenesis. Negative controls (without primary antibodies) have been added for a clearer visualization of the positive signal. **(A)** General view of the LG. Insets show the magnified region in **(B,C)**. Close-ups on the ductal region **(B)** and on the TEBs **(C)** reveal *Notch2* expression in the basal and suprabasal cell layers of the LG epithelium. Notch2 is also found in the mesenchymal compartment. Cleaved *Notch2* can be observed in the nuclei of cells in both basal and suprabasal cell layers at all the stages (**A-C**, white arrowheads) **(B,C)** Insets show the magnified region in the lower panels. Scale bars: **(A)** 50 μm; **(B,C)** upper panel: 5 μm; lower panel: 2 μm.

We used a transcriptomic analysis to investigate the expression levels of the Notch pathway elements at E18 and 34 weeks of age. This approach demonstrated an enrichment of various Notch pathway elements in embryonic LG. Interestingly, we found that *Notch2*, its ligand *Jagged1* (Shimizu et al., 1999) and its target gene *Hey1* (Rutenberg et al., 2006) were enriched during LG formation (Figures S7A,B). Quantitative PCR analysis confirmed the expression of *Notch2* and *Hey1* during LG morphogenesis from E15 to 34 weeks of age (Figure S7C), reflecting the involvement of Notch2 activity in forming LG, as well as in adult LG. We investigated Notch2 localization and activity by immunohistochemistry staining, targeting its intracellular domain (ICD), which is cleaved and translocated to the nucleus upon activation. We detected the cleaved Notch2 ICD in an increasing number of epithelial cells in both the TEB and the ductal territory from E16 to E18 (Figures 11A–C).

**Figure 12:**
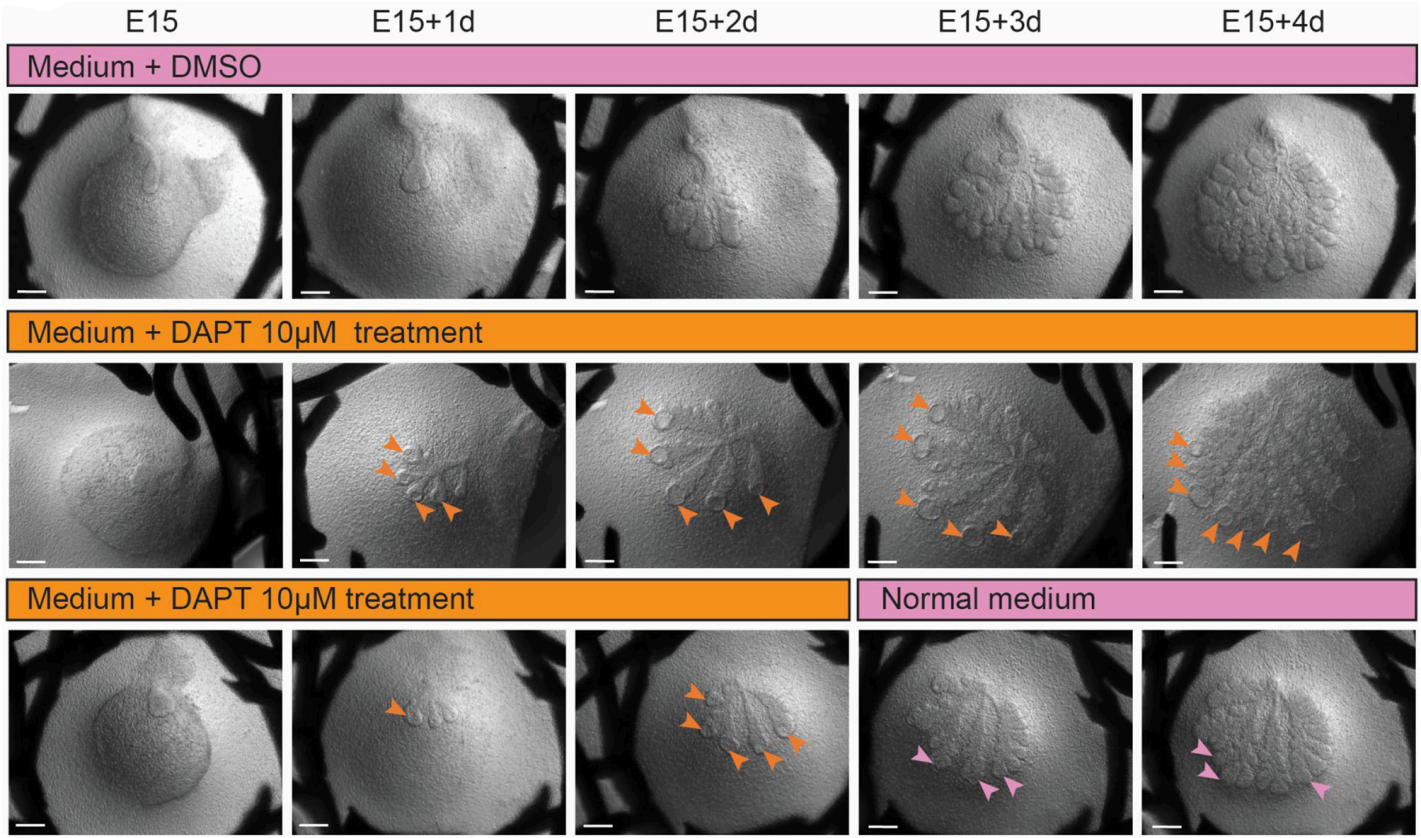
Inhibition of Notch pathway induces hollowed TEBs, which regenerate upon inhibition removal, and extra-branching, which is not rescued. *Ex vivo* cultures upon DAPT treatment (10 μM) from E15 to E15+4d. Upper panel shows DMSO-treated control. Hollowed TEBs (orange arrowheads) appear after 1 day of treatment (middle and lower panel) and are maintained throughout Notch inhibition. Upon DAPT removal, the TEBs morphology is rescued (pink arrowheads), but the extra branches phenotype is maintained. Scale bars: 200 μm.

To study further the contribution of Notch pathway in LG morphogenesis, we used a γ-secretase inhibitor (DAPT) in *ex vivo* cultures (Figure 12). While having an effect on off-targets (Zhao et al., 2008; Yoo et al., 2012), DAPT is a small molecule widely used to impair NICD cleavage, and consequently Notch activation (Jiang et al., 2011; Dvoriantchikova et al., 2017; Jing et al., 2017). We validated the efficiency of the inhibition by assessing the decrease of *Hey1* expression via qPCR (Figure S7D), and the drastic reduction of nuclear Notch2 ICD (Figure S7E). We observed that inhibition of the Notch pathway resulted in hollow TEBs already after 1 day of treatment (Figure 12). In line with previous report (Dvoriantchikova et al., 2017), we also observed extra-branching after 2 days of treatment. While the loss of TEB supra-basal cells, resulting from apoptosis as previously shown in salivary gland (Garcia-Gallastegui et al., 2014; Figure S8), was rescued promptly after DAPT treatment retrieval (Figure 12), the extra-branching phenotype remained.

**Figure 13:**
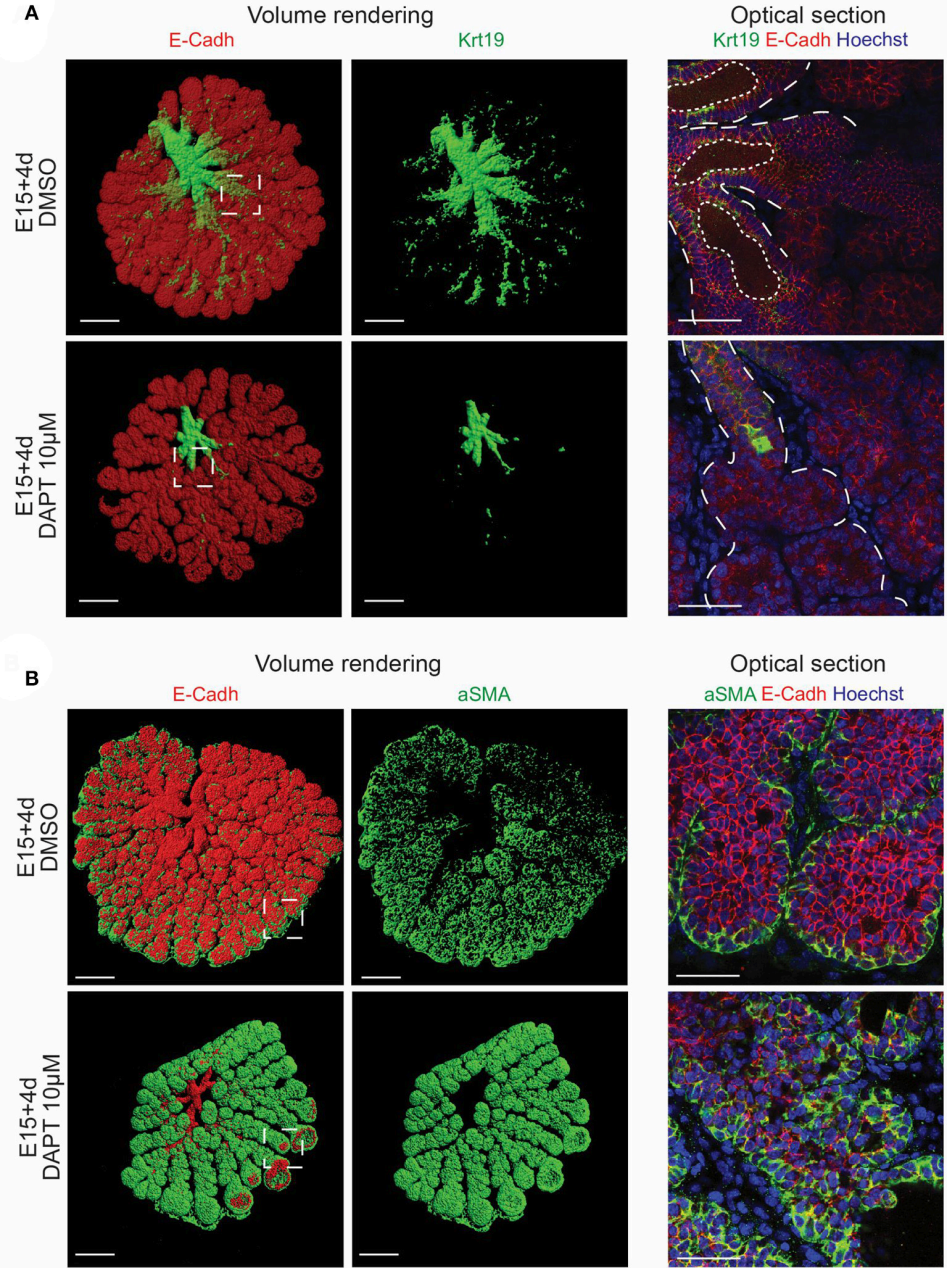
Inhibition of Notch pathway induces a switch from branch territory to TEB identity. **(A,B)** DAPT-treated samples after 4 days of treatment. **(A)** Volume renderings (left panel) and confocal images (right panel) of whole mount immunostaining for Krt19 and E-Cadherin reveal an arrest in the duct formation in the DAPT-treated samples in comparison to the DMSO control. Dotted lines highlights epithelial compartment and lumen. **(B)** Volume renderings (left panel) and confocal images (right panel) of whole mount immunostaining for aSMA and E-Cadherin demonstrate an enlargement of the *aSMA*+ domain in the DAPT-treated samples in comparison to the DMSO control. Krt19 and aSMA staining reflect an extension of the TEBs territory instead of ductal tree expansion. Scale bars: **(A,B)** left panel: 200 μm; right panel: 50 μm. Insets show the magnified region in the right panels.

To analyse the effect of Notch pathway on epithelium patterning, we investigated the localization of *Krt19*+ and *aSMA*+ territories in DAPT treated samples. Strikingly, the inhibition of Notch cleavage led to an arrest of LG tree formation (Figure 13A). We could not detect any Krt19+ cells in the branches, and the lumen did not form. Moreover, the aSMA domain expanded drastically, and was not anymore restricted to the TEBs. After inhibiting the Notch pathway for 5 days, aSMA was detected ectopically in the whole basal cell layer of the branches (Figure 13B). The increase of *aSMA* expression was confirmed by qPCR (Figure S9).

Our results suggested a crucial role of Notch pathway in enforcing the border between duct and TEB domain. Moreover, Notch activity seemed to be directly involved in the branch cell fate.

## Discussion

The formation and maturation of ectodermal organs are highly controlled processes, by which an ectoderm and a mesenchyme construct a structure fitting for a specific function. As an ectodermal appendage, LG secretes the aqueous component of the tear film to insure the maintenance of a healthy cornea. Because of the late eyelid opening in the mouse, the chronology of LG morphogenesis and maturation expands beyond the embryonic development, and continues postnatally. In our study, we used specific epithelial markers to decipher several mechanisms involved in LG formation and glandular tree expansion.

We discovered that Krt19 is an early marker of the presumptive ductal territory. During LG morphogenesis, Krt19 labeled the whole ductal tree. Finally, the *Krt19*+ cell population was found to be constituting the luminal domain of the mature LG. Prior to the first branching event, Krt19 domain seemed to co-localize with an accumulation of E-Cadherin at the membrane. E-Cadherin has previously been reported to play a key-role in cell-cell tension sensing and in the transduction of mechanical forces throughout epithelial compartment (for review, Sluysmans et al., 2017). Moreover, E-Cadherin is involved in the orientation of epithelial cell division, controlling tissue shapes (Hart et al., 2017). Our results indicated that E-Cadherin could play a role in the formation of a transient 3D spring-like structure within the epithelial bud that would participate to the sudden bud elongation, prior to the first branching event. We suggest that the cell-cell adhesion in the presumptive ductal domain is of utmost importance to insure the collective migration of the *Krt19*+ cells within the LG epithelium, as proposed in the mammary gland morphogenesis (Shamir and Ewald, 2015).

Our results pointed out the rapid decrease of cell proliferation in the ductal compartment during LG epithelial growth. Our data hinted toward a combination of apoptosis and epithelial reorganization to support the lumen formation and duct expansion in absence of proliferation. Taken together, it suggests a possible convergent extension process occurring in the ducts (for review, Keller et al., 2008). A similar phenomenon was previously described in the renal tubular formation (Castelli et al., 2013), as well as in the salivary gland duct formation in the Drosophila (Girdler and Roper, 2014).

We confirmed the expression of *Krt14* and *aSMA* in different epithelial compartments of forming LG. While Krt14 marked indistinctly the branch and TEB domains, *aSMA* expression outlined the TEB domain in early stages, highlighting a fate difference between branch and TEB epithelial cells. Interestingly, we showed that the number of TEBs was remarkably predictable at each stage of embryonic LG development. In contrast, the branching pattern, reflected by the position of the bifurcations, did not seem to follow a predetermined scheme.

A recent study stated the development of extra-branches in the LG upon Notch inhibition (Dvoriantchikova et al., 2017). The use of specific markers allowed us to propose a different conclusion. Strikingly, the inhibition of Notch signaling induced the ectopic formation of *aSMA*+ TEB domains along the LG branches. Therefore, we propose that ductal cells retain a degree of plasticity, enabling the switch from duct to TEB cell identity, as previously reported during the kidney morphogenesis (Watanabe and Costantini, 2004). Lateral branch development requires cell specification, involving an identity switch from ductal to TEB cells, allowing the extension of a new branch from the newly specified TEB cells (for review, Costantini and Kopan, 2010). We hypothesize that Notch activity helps maintaining the branch identity until a possible mesenchymal signal would induce the formation of a lateral branch.

A previous report on lung development demonstrated the implication of Notch signaling in proximal structure formation and distal progenitors expansion, along with the expansion of FGF10 expression domain in the mesenchyme at the extra-branching sites (Tsao et al., 2008). Accordingly, we showed that Notch signaling was required for TEB maintenance, correct expansion and *ipso facto* for LG epithelium integrity. Interestingly, Alagille syndrome (ALGS) is a genetic disease resulting from *Notch2* and/or *Jagged1* mutation (Turnpenny and Ellard, 2012). Among the associated symptoms, corneal pannus and other ophthalmologic defects have been reported in ALGS patients (Hingorani et al., 1999). Moreover, corneal pannus has previously been linked to severe DED appearing secondary to untreated ocular defect (Sivaraman et al., 2016). Combining these observations to our results, we hypothesize that the modification of Notch signaling resulting from the genetic mutation in ALGS may lead to LG morphological defects, subsequently inducing a dry eye dependent corneal pannus, as reported in ALGS patients.

Collectively, our results on LG morphogenesis and late maturation displayed already known mechanisms involved in other gland formation. Similarly to the salivary gland (Nedvetsky et al., 2014), we showed that the LG duct lumen forms by luminal cells apoptosis. Additionally, we proposed that LG tree branching occurs in a stochastic manner, comparable to the mammary gland morphogenesis (for review, Sternlicht, 2006). Furthermore, our results on the inhibition of Notch pathway were similar to the DAPT effect on salivary gland and lung development (Tsao et al., 2008; Dvoriantchikova et al., 2017). Therefore, LG singularity compared to other glands comes from its function of secreting a fluid nourishing and protecting the eye surface (Conrady et al., 2016), which could result from the early expression of *Pax6* in the LG epithelium, as suggested previously (Makarenkova et al., 2000). Taken together, LG morphogenesis recapitulates developmental processes of other branched organs, making this gland a fitting model to study glandular development.

## Author contributions

AK carried out the experiments. AK and FM designed the experiments, analyzed the data, wrote the manuscript.

### Conflict of interest statement

The authors declare that the research was conducted in the absence of any commercial or financial relationships that could be construed as a potential conflict of interest.

## Supplementary material

Figure S1 *aSMA, Krt14, Krt19* are expressed in embryonic and postnatal LG. **(A)** *aSMA* and *Krt14* qPCR analysis from E15 to adult reports an overall increase in both genes expression during LG development. **(B)** *Krt19* qPCR analysis reveals an increase in *Krt19* expression from E15 to adult, with a peak of expression at E18, and a diminution in the adult stage. Gene expression levels were normalized to *GAPDH* expression.

Figure S2 aSMA-Cre does not allow the genetic fate mapping of *aSMA*+ cells progeny in embryonic lacrimal gland. **(A)** Is an overview and **(B)** a close-up of dissected out E18 aSMA-Cre;R26R-mTmG LG. Although, *aSMA* expression pattern showed positive cells in the basal layer of all the TEBs (Figure 3), only few *aSMA*+ cells were observed in one of the TEBs with the aSMA-Cre recombination. Scale bars: **(A)** 200 μm; **(B)** 50 μm.

Figure S3 LG postnatal maturation. **(A)** Immunohistochemistry staining on LG paraffin sections from P7 to P50. Optical sections of confocal images show E-cadherin, aSMA and Krt14 localization. E-Cadherin is detected in all epithelial cells, and allows the visualization of the epithelium compaction occurring from P13 onwards. *aSMA* and *Krt14* expression patterns show a continuous organization process in postnatal stages. *aSMA*+ and *Krt14*+ cells density decreases along with LG postnatal maturation. Both markers are expressed in the MECs. In addition, *aSMA* is expressed in small clusters of cells (white arrowheads) and *Krt14* in the external layer of the ducts (orange arrowheads). **(B)** RT-PCR analysis of E15 to P50 LGs demonstrates *Aquaporin1, 5* and *8* dynamic expression profiles. *Aquaporin1* is expressed in all except the latest studied stage. *Aquaporin5* is found in all the stages and *Aquaporin8* only in the postnatal stages. Scale bars: **(A)** 20 μm.

Figure S4 Strategy used for the Confetti reporter mouse. **(A)** The Confetti genetic construct scheme was adapted from Snippert et al. (2010). The first recombination event allows to sort cells that are able to express *nGFP* and/or *YFP* from cells that can express *RFP* and/or *mCFP*. **(B)** Confocal images give an example of single-colored (purple arrowheads) and double-colored cells (white arrowheads) upon continuous recombination. **(C)** After the first recombination event, cells can be sorted into two domains: *GFP* and/or *YFP* expressing cells are regrouped in the orange cell domain, and *RFP* and/or *CFP* expressing cells are regrouped in the violet cell domain. Scale bars: **(B)** 20 μm.

Figure S5 K14-Cre43 crossed with reporter mouse lines reveals *Krt14* negative cells in LG epithelial compartment. **(A)** Optical section of K14-Cre43;R26R-Tdtomato LG at E17 shows unlabeled cells in the epithelial compartment (white arrowheads). Dotted lines delimitate epithelial regions. **(B)** Confocal images of whole mount for Krt14 on K14-Cre43;R26R-mTmG LGs from E16 to E18. Optical sections show *Krt14* negative (and non-recombined, red) cells in the epithelial compartment. Dotted lines delimitate *Krt14* negative cells. Scale bars: **(A)** 30 μm; **(B)** 20 μm.

Figure S6 Krt19 localization remains in the duct luminal cells in postnatal LG. Immunohistochemistry staining on LG paraffin sections from P7 to P50. Optical sections of confocal images show *Krt14* and *Krt19* expression patterns. *Krt14* is expressed in the basal layer of the ducts (orange arrowheads). Krt19 remains localized in the luminal side of the ducts (white arrowheads). Scale bars: 20 μm.

Figure S7 A transcriptomic analysis revealed an enrichment of Notch pathway elements in embryonic LG. **(A)** Heat map showing the different Notch pathway elements enrichment in embryonic compared to adult (34 weeks of age) LG. *Notch2, Hey1* and *Jagged1* fold changes and *p*-values are summarized in the table **(B)**. **(C)** *Notch2* and *Hey1* qPCR analysis revealed modifications in both genes expression during LG development from E15 to adult. *Notch2* relative expression was higher in the adult stage in comparison to the embryonic stages. *Hey1* relative expression shows a peak at E16, but no difference between E18 and the adult stage. **(D)** *Hey1* relative expression was used to assess Notch inhibition efficiency in DAPT-treated *ex vivo* cultures. *Hey1* expression was reduced to 20% upon DAPT treatment. Gene expression levels were normalized to *GAPDH* expression for subsequent qPCR analysis. **(E)** Immunohistochemistry staining for Cleaved-Notch2 at E15+2d are used to assess Notch inhibition efficiency. Negative controls (without primary antibodies) have been added for a clearer visualization of the positive signal. Cleaved-Notch2 can be observed in the DMSO control sample, but not in the DAPT-treated sample. Scale bars: **(E)** 4 μm.

Figure S8 Notch pathway inhibition induces TEB suprabasal cell loss by apoptosis. **(A)** Haematoxylin-Eosin staining of DAPT-treated samples reveal empty TEBs after 4 days of culture. **(B,C)** Optical sections for Cleaved-Caspase 3 whole mount immunostaining reveal apoptotic cells in the TEBs of DAPT-treated samples after 1 day of treatment (white arrowheads). Insets in **(B)** show the magnified region in **(C)**. Scale bars: **(A,B)** 100μm; **(C)** 30μm.

Figure S9 *aSMA* expression increases upon Notch pathway inhibition. Quantitative PCR analysis shows the relative expression of *aSMA* (normalized to *GAPDH* expression levels). *aSMA* expression level increases after 2 and 3 days of DAPT treatment, in comparison to the control. ^*^*p* < 0.01 was considered as statistically significant (Student's t-test). Error bars represent standard deviations (Biological and technical triplicates were analyzed per time point).

Movie 1 Krt19 localization reveals a 3D spring-like shape in the *ex vivo* culture E15 LG. Surface and volume rendering of Krt19 whole mount immunohistochemistry staining from E15 LG *ex vivo* culture. Hoechst staining was used to create the surface rendering allowing the visualization of the whole epithelial bud (white).

Movie 2 Krt19 localization reveals a 3D spring-like shape in the *in vivo* E15 LG. Surface and volume rendering of Krt19 whole mount immunohistochemistry staining from *in vivo* E15 LG. Hoechst staining was used to create the surface rendering allowing the visualization of the whole epithelial bud (white).
