# Supplementary figures and images for: Epithelial Markers aSMA, Krt14, and Krt19 Unveil Elements of Murine Lacrimal Gland Morphogenesis and Maturation
Source: Front Physiol. 2017 Sep 26;8:739. doi: 10.3389/fphys.2017.00739 (PMC5627580; doi:10.3389/fphys.2017.00739)

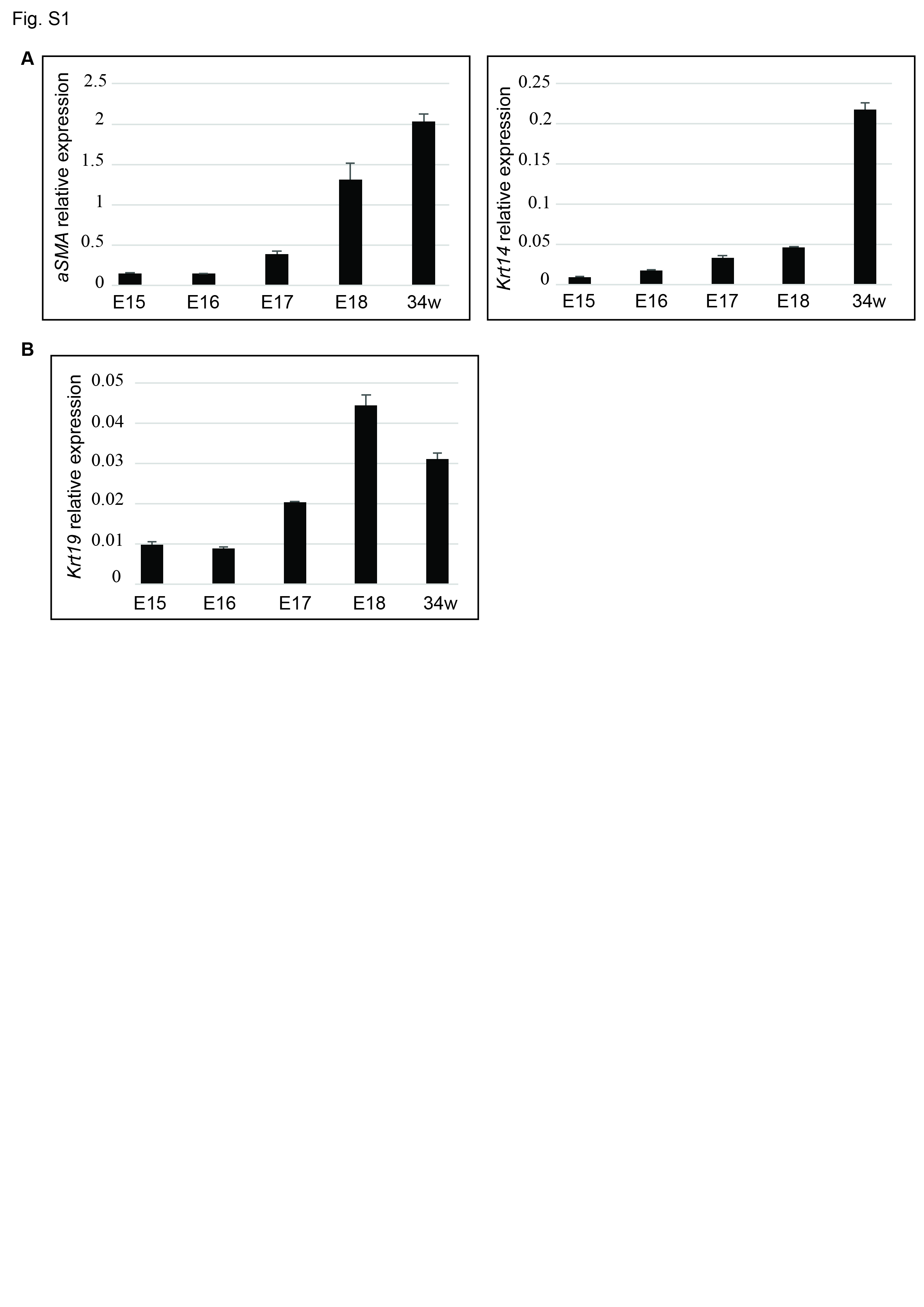

Supplement: Figure S1 — aSMA, Krt14, Krt19 are expressed in embryonic and postnatal LG. (A) aSMA and Krt14 qPCR analysis from E15 to adult reports an overall increase in both genes expression during LG development. (B) Krt19 qPCR analysis reveals an increase in Krt19 expression from E15 to adult, with a peak of expression at E18, and a diminution in the adult stage. Gene expression levels were normalized to GAPDH expression. [file Image1.JPEG]

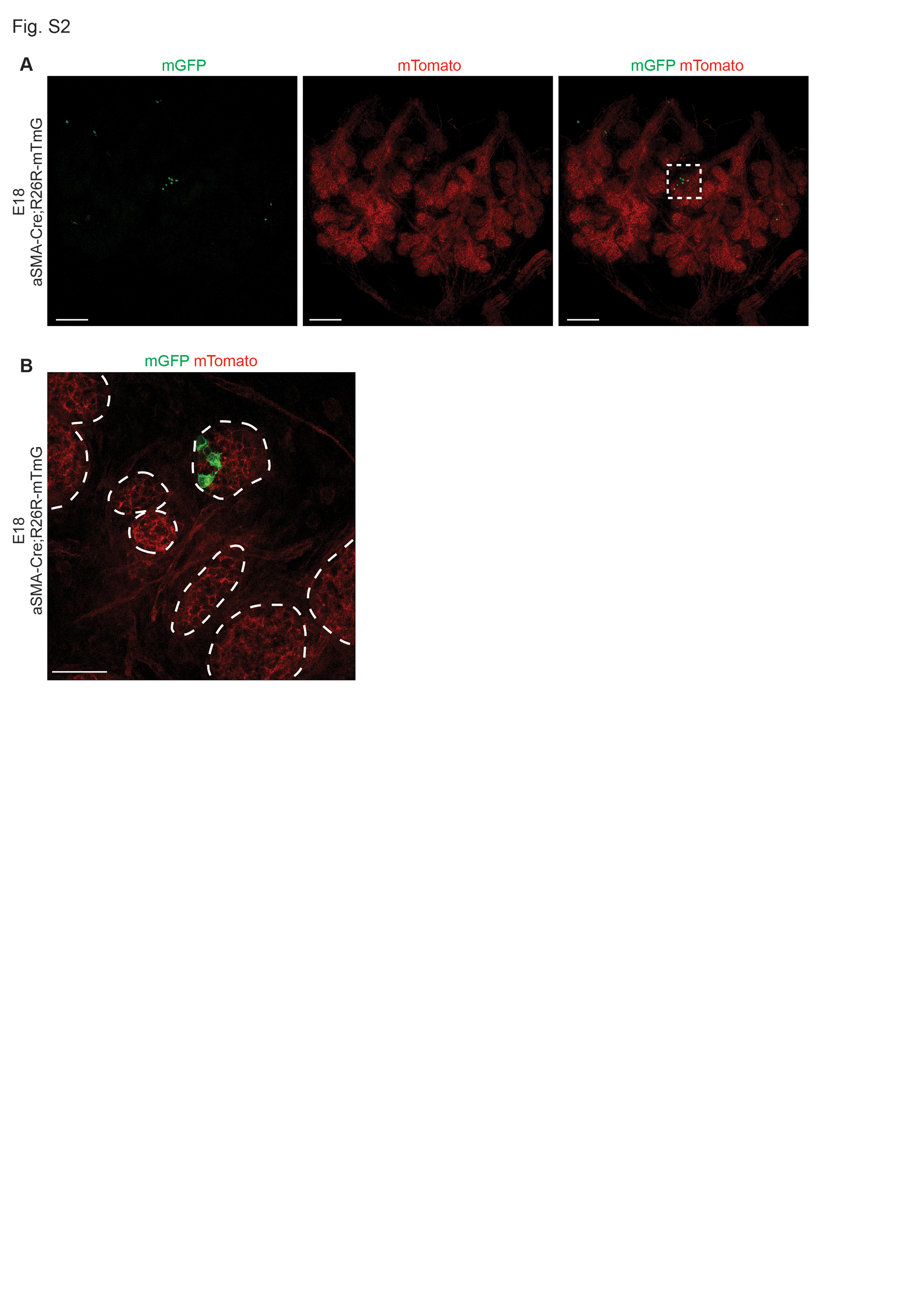

Supplement: Figure S2 — aSMA-Cre does not allow the genetic fate mapping of aSMA+ cells progeny in embryonic lacrimal gland. (A) Is an overview and (B) a close-up of dissected out E18 aSMA-Cre;R26R-mTmG LG. Although, aSMA expression pattern showed positive cells in the basal layer of all the TEBs (Figure 3), only few aSMA+ cells were observed in one of the TEBs with the aSMA-Cre recombination. Scale bars: (A) 200 μm; (B) 50 μm. [file Image2.JPEG]

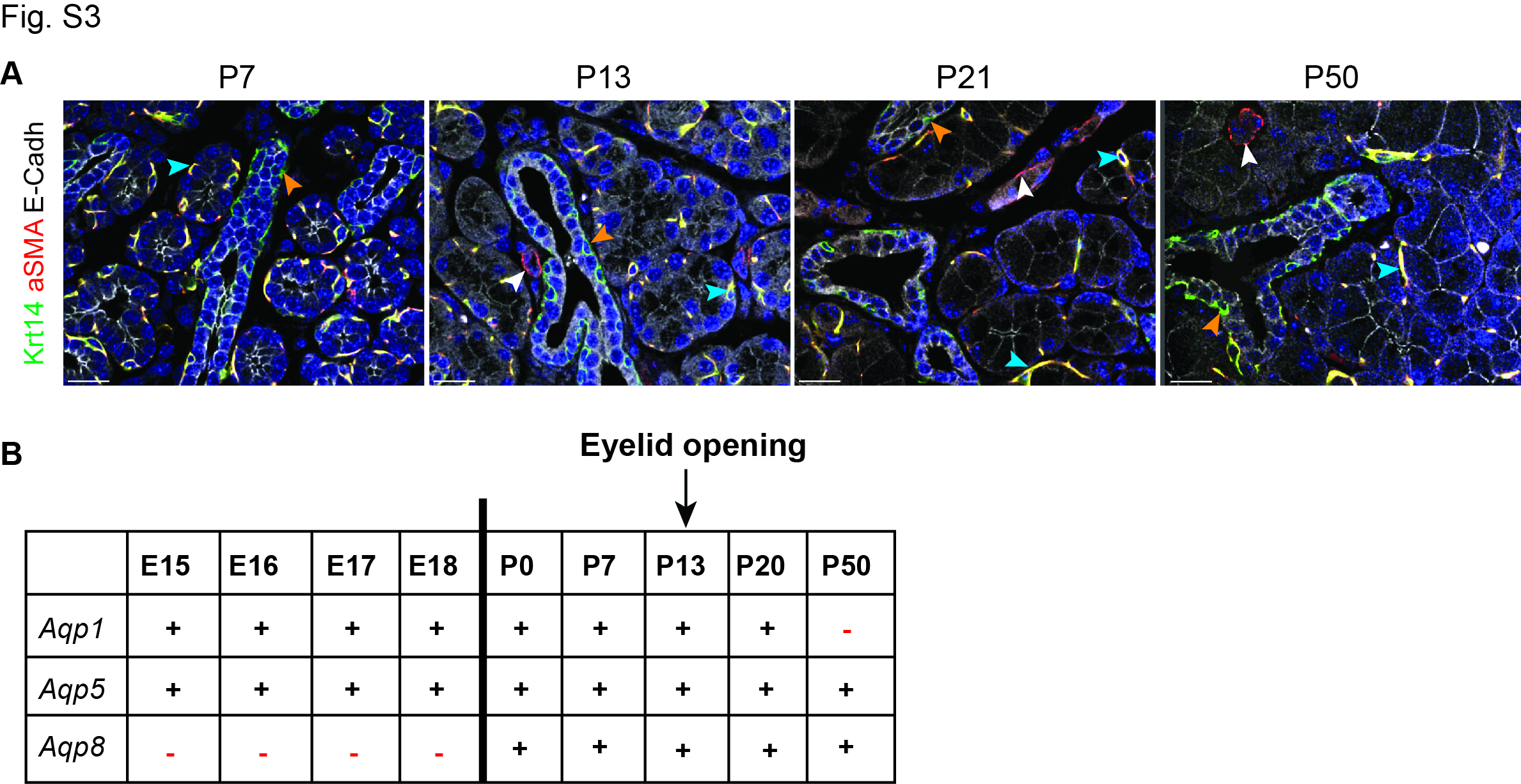

Supplement: Figure S3 — LG postnatal maturation. (A) Immunohistochemistry staining on LG paraffin sections from P7 to P50. Optical sections of confocal images show E-cadherin, aSMA and Krt14 localization. E-Cadherin is detected in all epithelial cells, and allows the visualization of the epithelium compaction occurring from P13 onwards. aSMA and Krt14 expression patterns show a continuous organization process in postnatal stages. aSMA+ and Krt14+ cells density decreases along with LG postnatal maturation. Both markers are expressed in the MECs. In addition, aSMA is expressed in small clusters of cells (white arrowheads) and Krt14 in the external layer of the ducts (orange arrowheads). (B) RT-PCR analysis of E15 to P50 LGs demonstrates Aquaporin1, 5 and 8 dynamic expression profiles. Aquaporin1 is expressed in all except the latest studied stage. Aquaporin5 is found in all the stages and Aquaporin8 only in the postnatal stages. Scale bars: (A) 20 μm. [file Image3.JPEG]

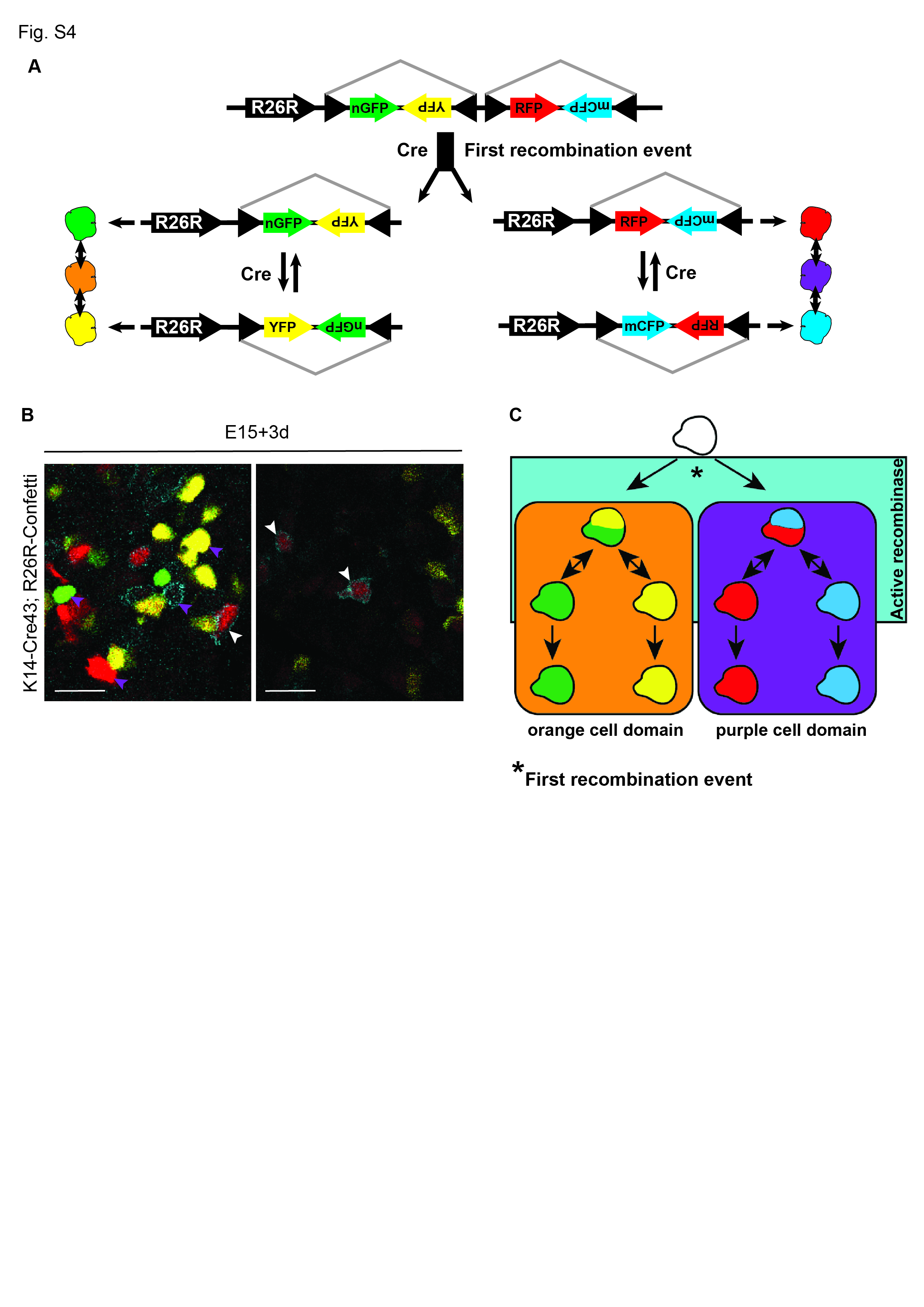

Supplement: Figure S4 — Strategy used for the Confetti reporter mouse. (A) The Confetti genetic construct scheme was adapted from Snippert et al. (2010). The first recombination event allows to sort cells that are able to express nGFP and/or YFP from cells that can express RFP and/or mCFP. (B) Confocal images give an example of single-colored (purple arrowheads) and double-colored cells (white arrowheads) upon continuous recombination. (C) After the first recombination event, cells can be sorted into two domains: GFP and/or YFP expressing cells are regrouped in the orange cell domain, and RFP and/or CFP expressing cells are regrouped in the violet cell domain. Scale bars: (B) 20 μm. [file Image4.JPEG]

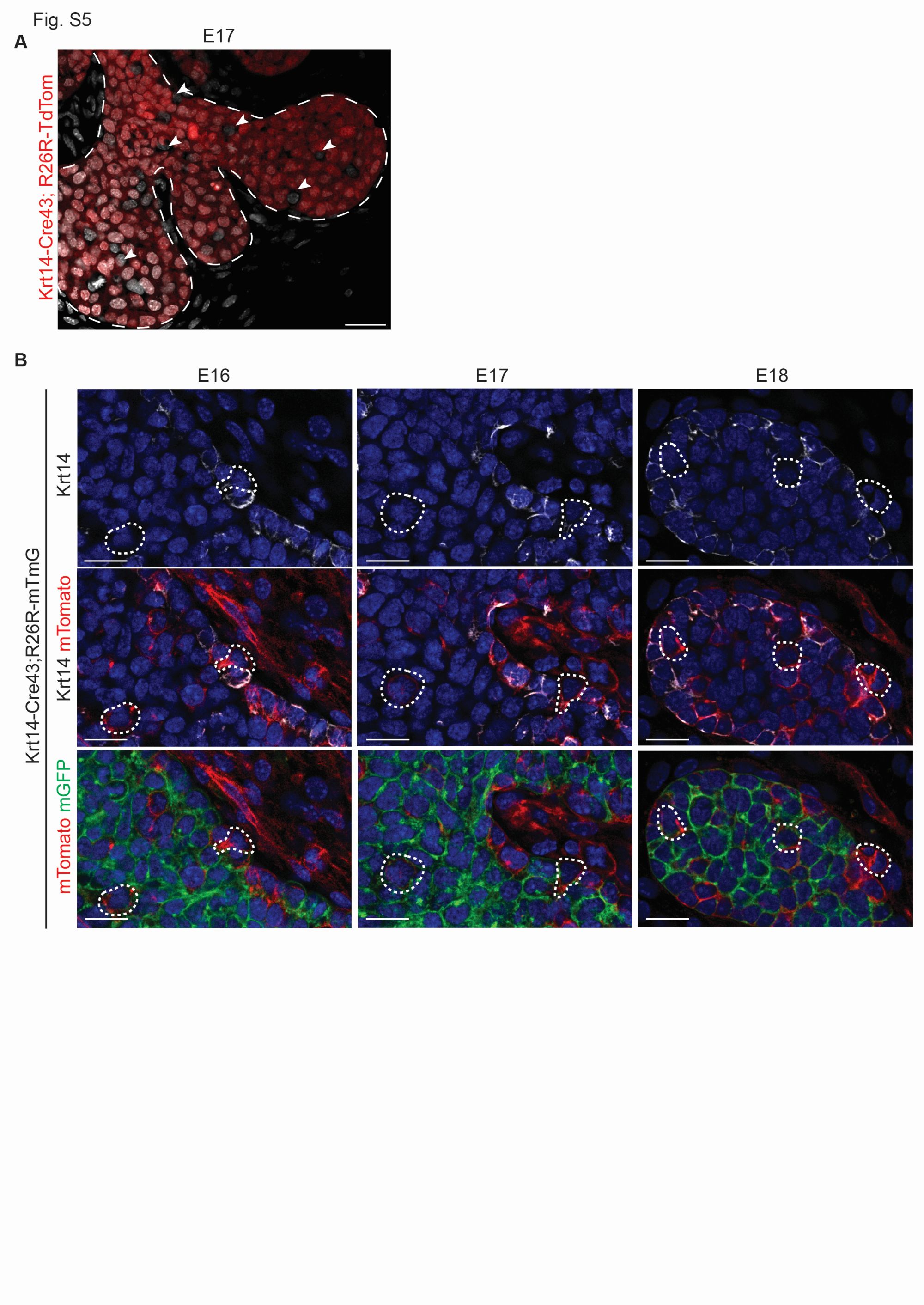

Supplement: Figure S5 — K14-Cre43 crossed with reporter mouse lines reveals Krt14 negative cells in LG epithelial compartment. (A) Optical section of K14-Cre43;R26R-Tdtomato LG at E17 shows unlabeled cells in the epithelial compartment (white arrowheads). Dotted lines delimitate epithelial regions. (B) Confocal images of whole mount for Krt14 on K14-Cre43;R26R-mTmG LGs from E16 to E18. Optical sections show Krt14 negative (and non-recombined, red) cells in the epithelial compartment. Dotted lines delimitate Krt14 negative cells. Scale bars: (A) 30 μm; (B) 20 μm. [file Image5.JPEG]

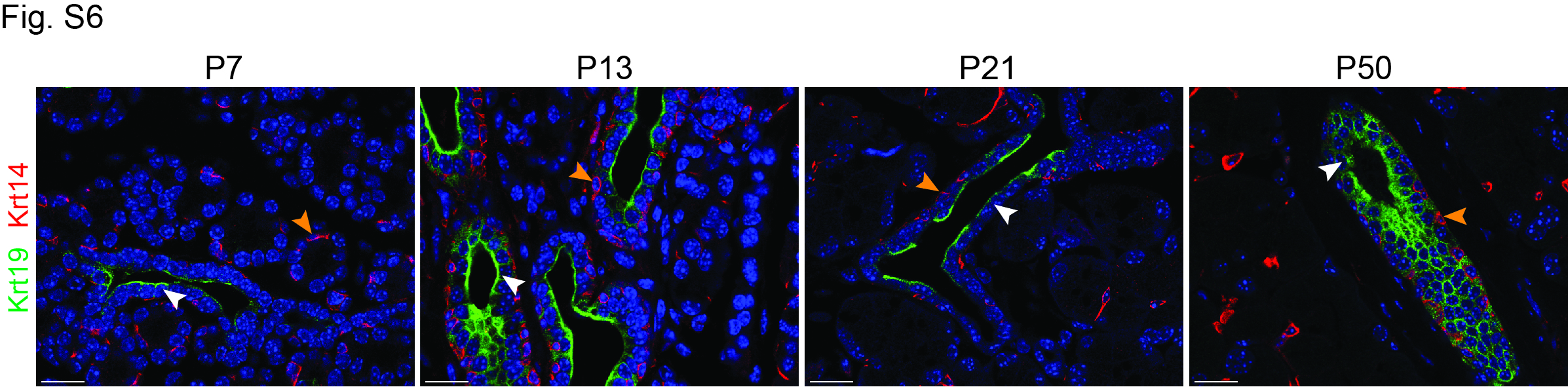

Supplement: Figure S6 — Krt19 localization remains in the duct luminal cells in postnatal LG. Immunohistochemistry staining on LG paraffin sections from P7 to P50. Optical sections of confocal images show Krt14 and Krt19 expression patterns. Krt14 is expressed in the basal layer of the ducts (orange arrowheads). Krt19 remains localized in the luminal side of the ducts (white arrowheads). Scale bars: 20 μm. [file Image6.JPEG]

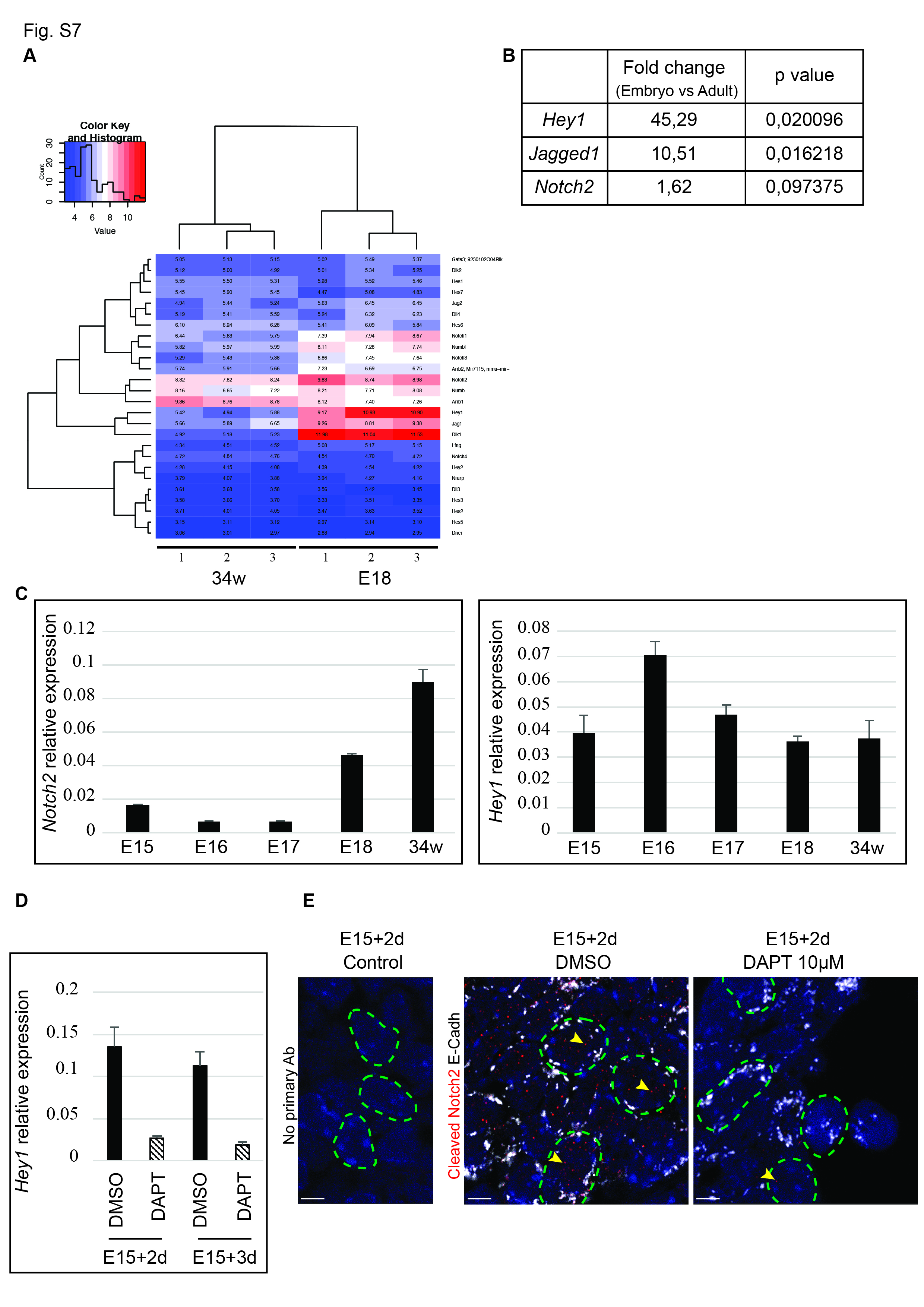

Supplement: Figure S7 — A transcriptomic analysis revealed an enrichment of Notch pathway elements in embryonic LG. (A) Heat map showing the different Notch pathway elements enrichment in embryonic compared to adult (34 weeks of age) LG. Notch2, Hey1 and Jagged1 fold changes and p-values are summarized in the table (B). (C) Notch2 and Hey1 qPCR analysis revealed modifications in both genes expression during LG development from E15 to adult. Notch2 relative expression was higher in the adult stage in comparison to the embryonic stages. Hey1 relative expression shows a peak at E16, but no difference between E18 and the adult stage. (D) Hey1 relative expression was used to assess Notch inhibition efficiency in DAPT-treated ex vivo cultures. Hey1 expression was reduced to 20% upon DAPT treatment. Gene expression levels were normalized to GAPDH expression for subsequent qPCR analysis. (E) Immunohistochemistry staining for Cleaved-Notch2 at E15+2d are used to assess Notch inhibition efficiency. Negative controls (without primary antibodies) have been added for a clearer visualization of the positive signal. Cleaved-Notch2 can be observed in the DMSO control sample, but not in the DAPT-treated sample. Scale bars: (E) 4 μm. [file Image7.JPEG]

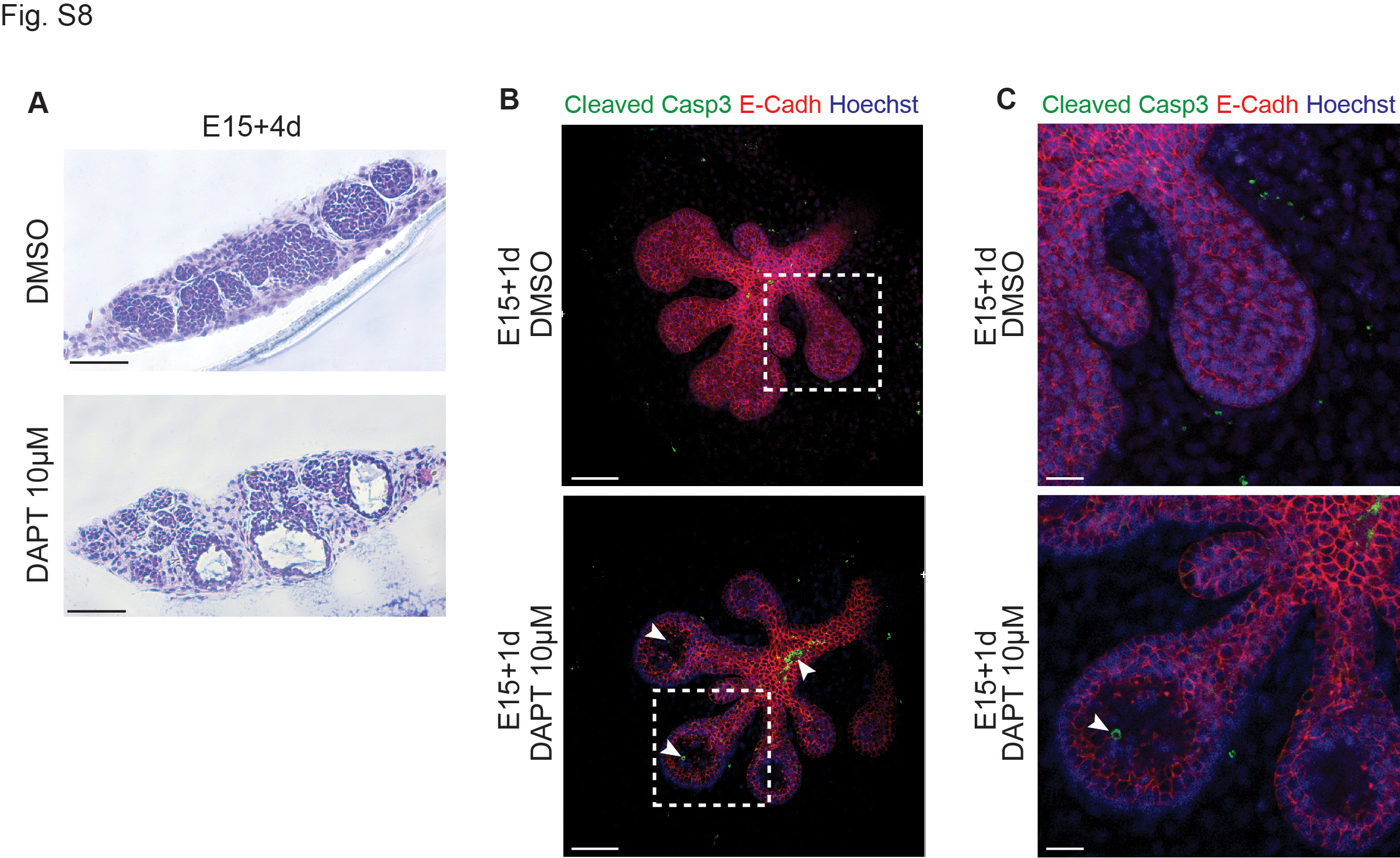

Supplement: Figure S8 — Notch pathway inhibition induces TEB suprabasal cell loss by apoptosis. (A) Haematoxylin-Eosin staining of DAPT-treated samples reveal empty TEBs after 4 days of culture. (B,C) Optical sections for Cleaved-Caspase 3 whole mount immunostaining reveal apoptotic cells in the TEBs of DAPT-treated samples after 1 day of treatment (white arrowheads). Insets in (B) show the magnified region in (C). Scale bars: (A,B) 100μm; (C) 30μm. [file Image8.JPEG]

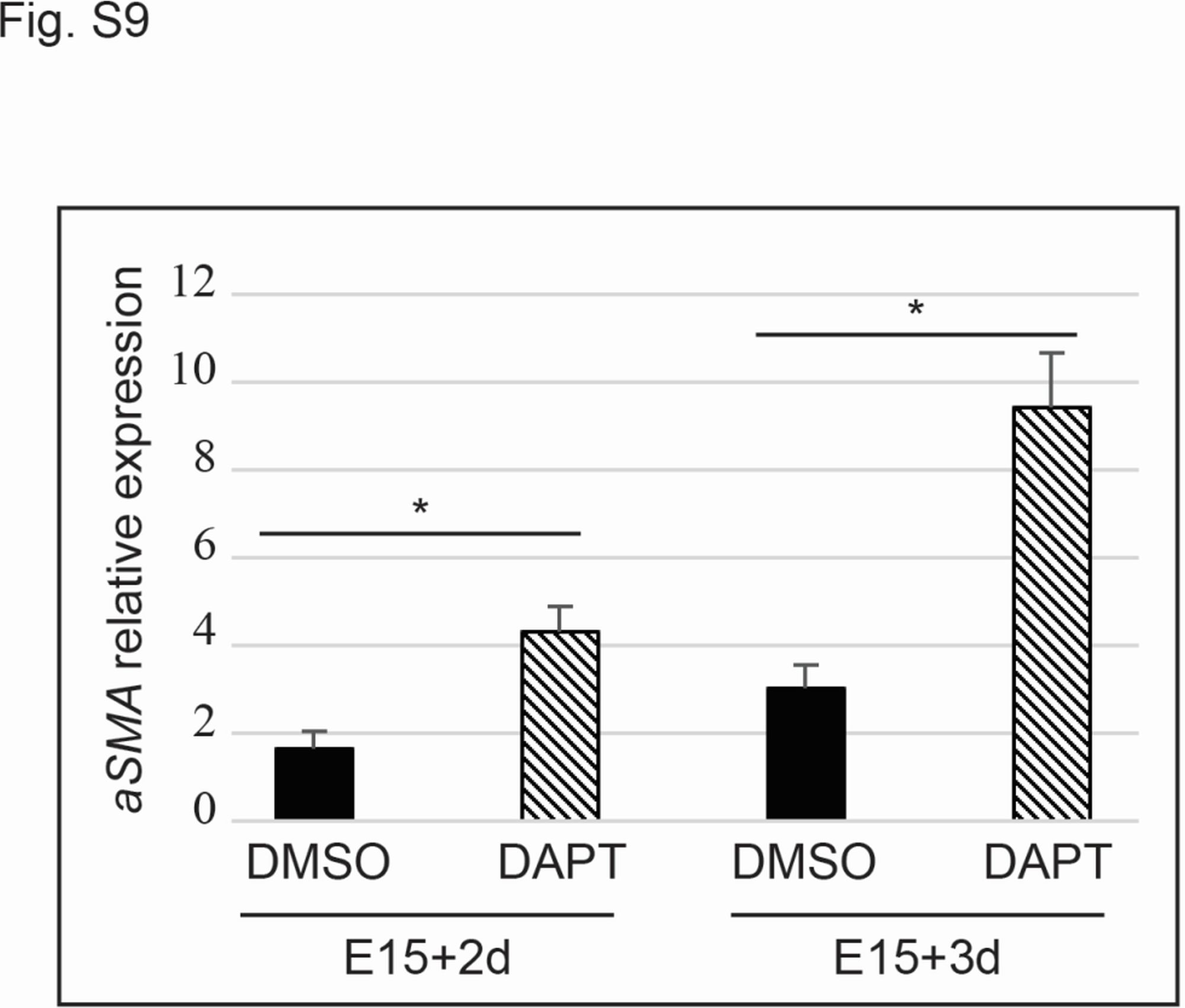

Supplement: Figure S9 — aSMA expression increases upon Notch pathway inhibition. Quantitative PCR analysis shows the relative expression of aSMA (normalized to GAPDH expression levels). aSMA expression level increases after 2 and 3 days of DAPT treatment, in comparison to the control. *p < 0.01 was considered as statistically significant (Student's t-test). Error bars represent standard deviations (Biological and technical triplicates were analyzed per time point). [file Image9.JPEG]
